# Supplementary material for: How single mutations affect viral escape from broad and narrow antibodies to H1 influenza hemagglutinin
Source: Nat Commun. 2018 Apr 11;9:1386. doi: 10.1038/s41467-018-03665-3 (PMC5895760; doi:10.1038/s41467-018-03665-3)
Supplement: Supplementary file 1 — Supplementary Information(PDF 1400 kb) [file 41467_2018_3665_MOESM1_ESM.pdf]

Supplementary Information

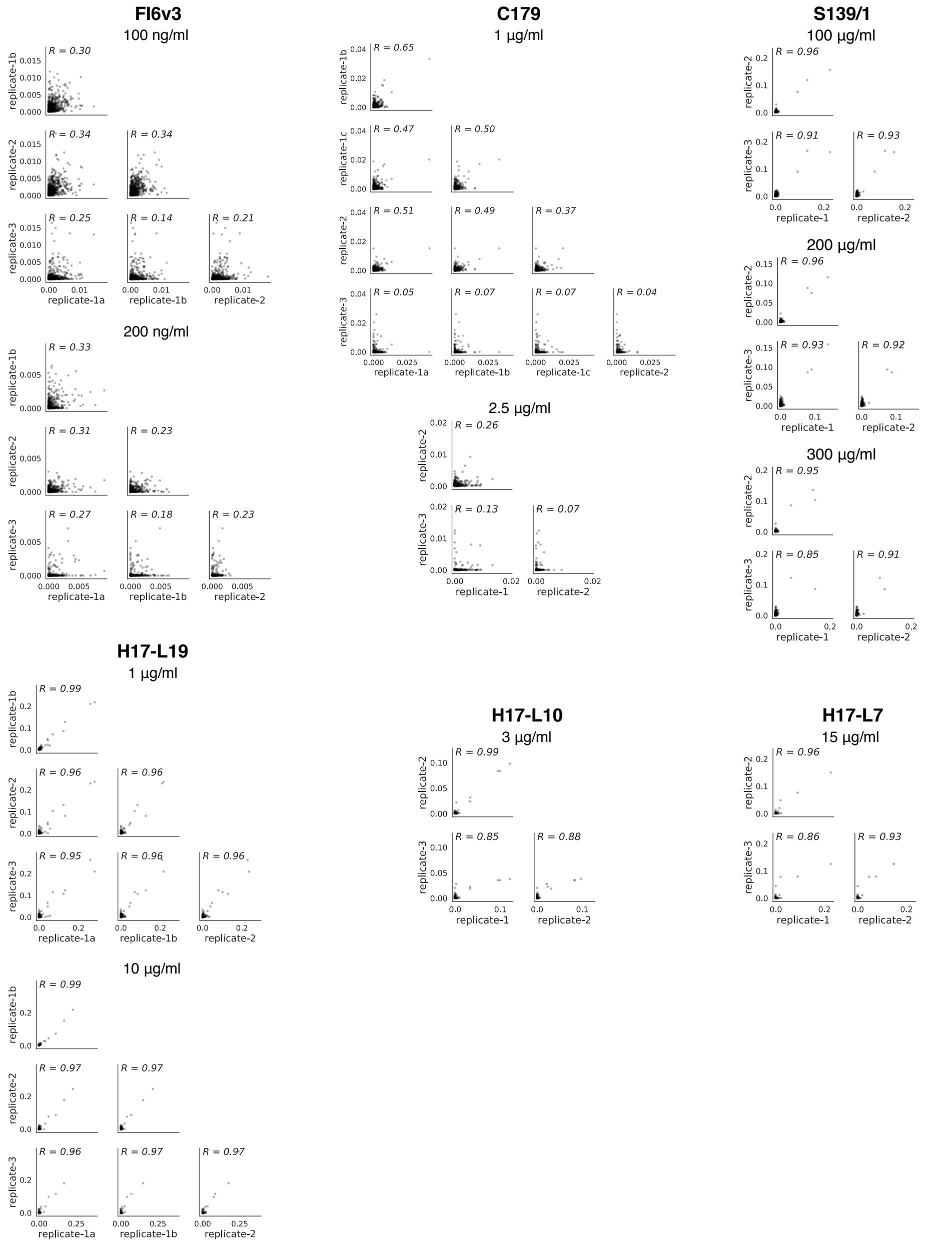

Supplementary Fig. 1: Correlations across experimental replicates. Each point represents one site in HA, and gives the fraction surviving above average across all amino-acid mutations at that site, as calculated using Equation 10. The replicates are highly correlated for antibodies with strong escape mutations (S139/1, H17-L19, H17-L10, and H17-L7), and reasonably correlated for antibodies with only weak escape mutations (FI6v3 and C179).

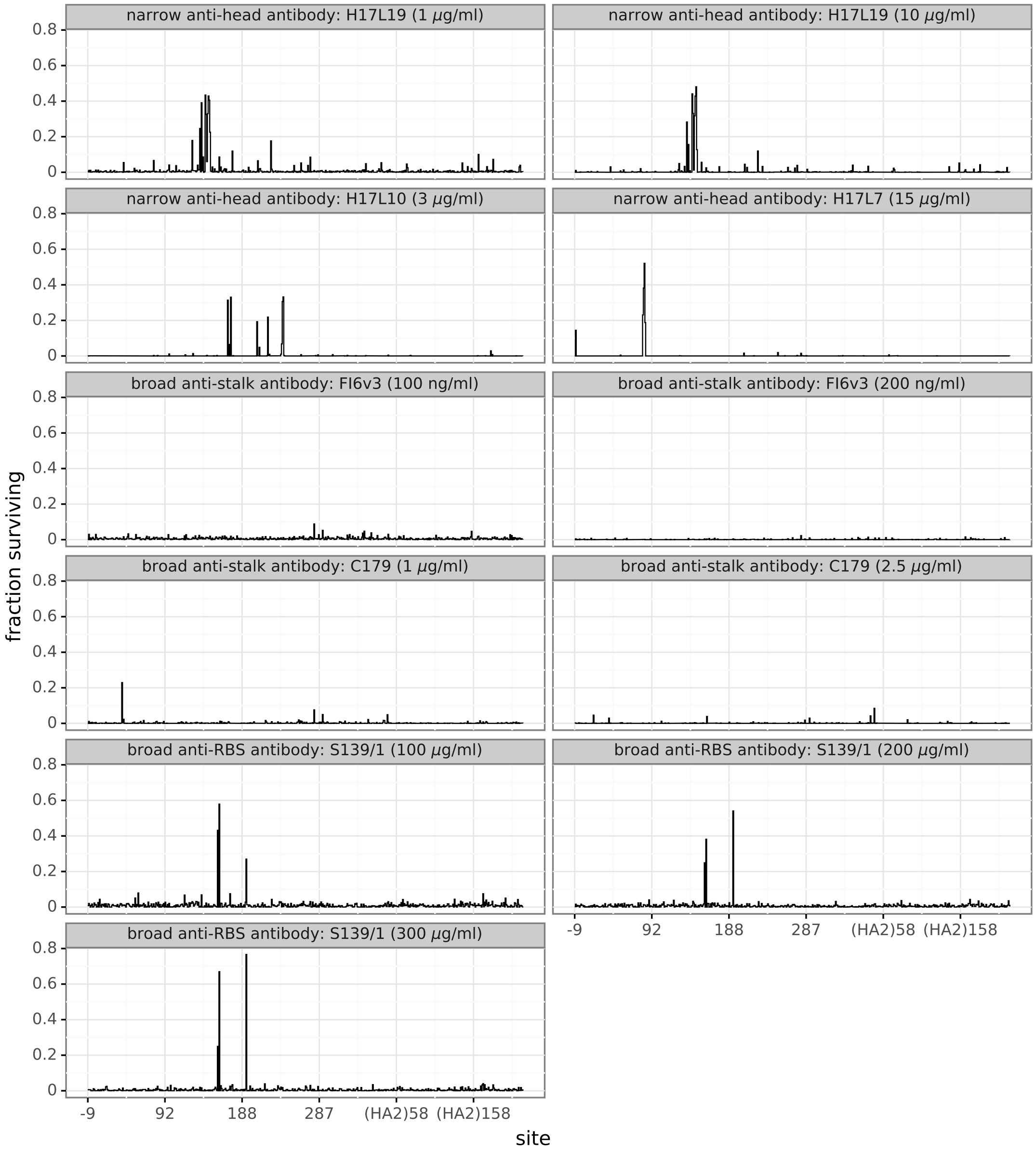

Supplementary Fig. 2: The excess fraction surviving for the single strongest escape mutation at each site. This plot differs from Figure 4 in that the height of the line indicates the excess fraction of virions that survive the antibody selection for the single strongest escape mutation at that site, rather than the average across all amino-acid mutations at that site.

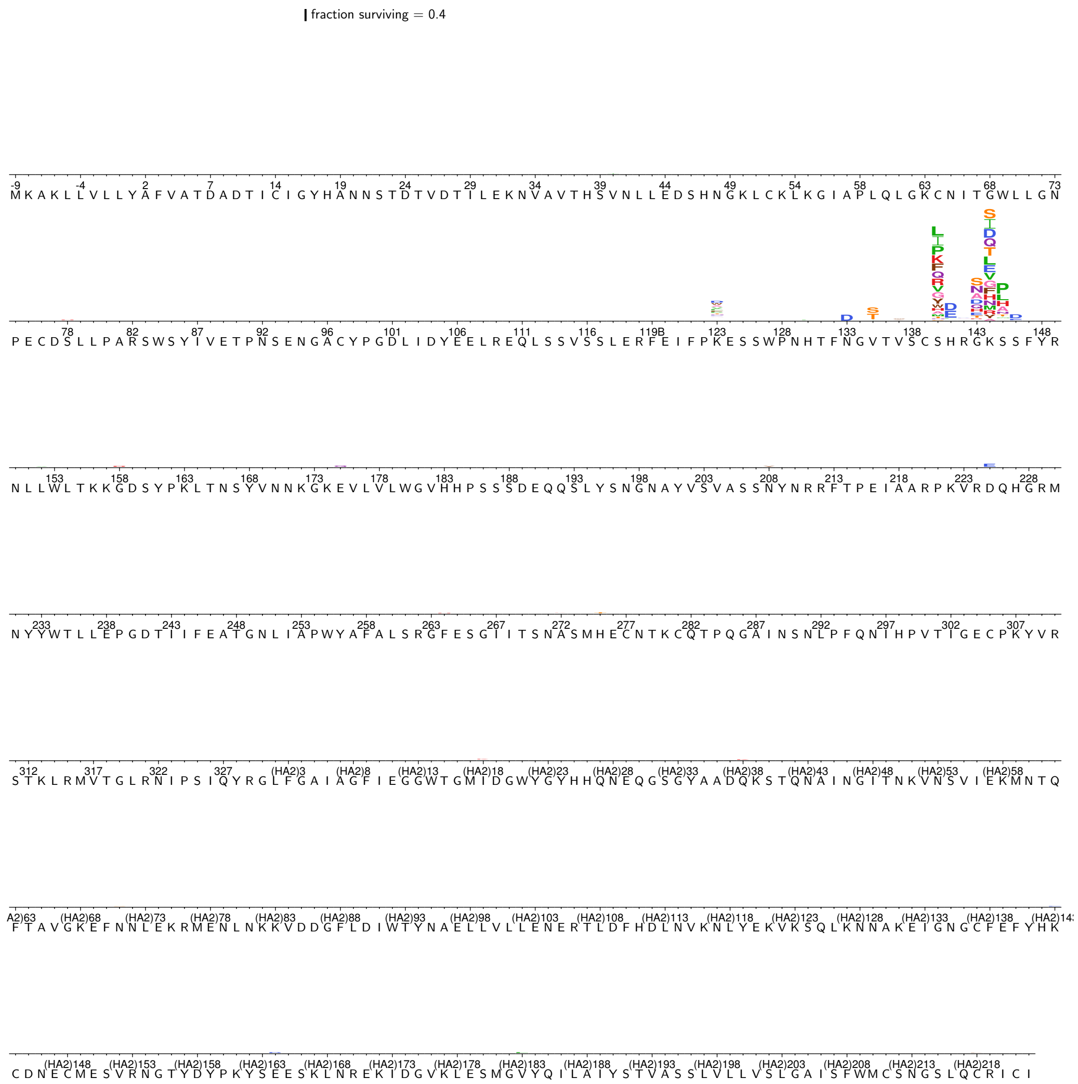

Supplementary Fig. 3: The excess fraction surviving selection with antibody H17L19 for all amino-acid mutations. The excess fraction surviving for each replicate was computed using Equation 2, then we took the median across all technical and biological replicates for each antibody concentration, and then took the medians of those values across concentrations. The height of each letter is proportional to the excess fraction surviving of virions with that mutation. The scale bar at the top of the plot relates the letter heights to the actual fractions. The sites are labeled using H3 numbering.

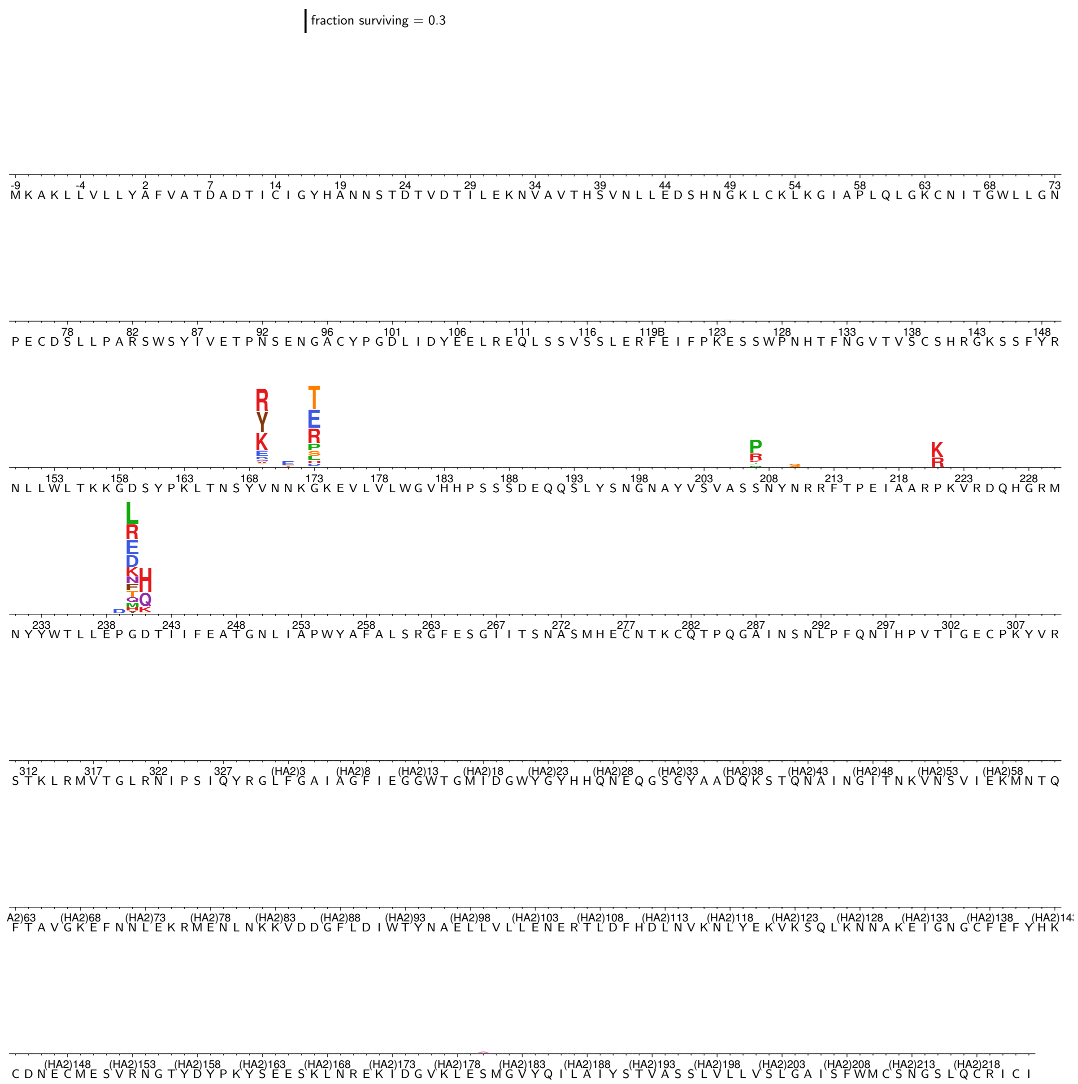

Supplementary Fig. 4: The excess fraction surviving selection with antibody H17L10 for all amino-acid mutations. The excess fraction surviving for each replicate was computed using Equation 2, then we took the median across all technical and biological replicates for each antibody concentration, and then took the medians of those values across concentrations. The height of each letter is proportional to the excess fraction surviving of virions with that mutation. The scale bar at the top of the plot relates the letter heights to the actual fractions. The sites are labeled using H3 numbering.

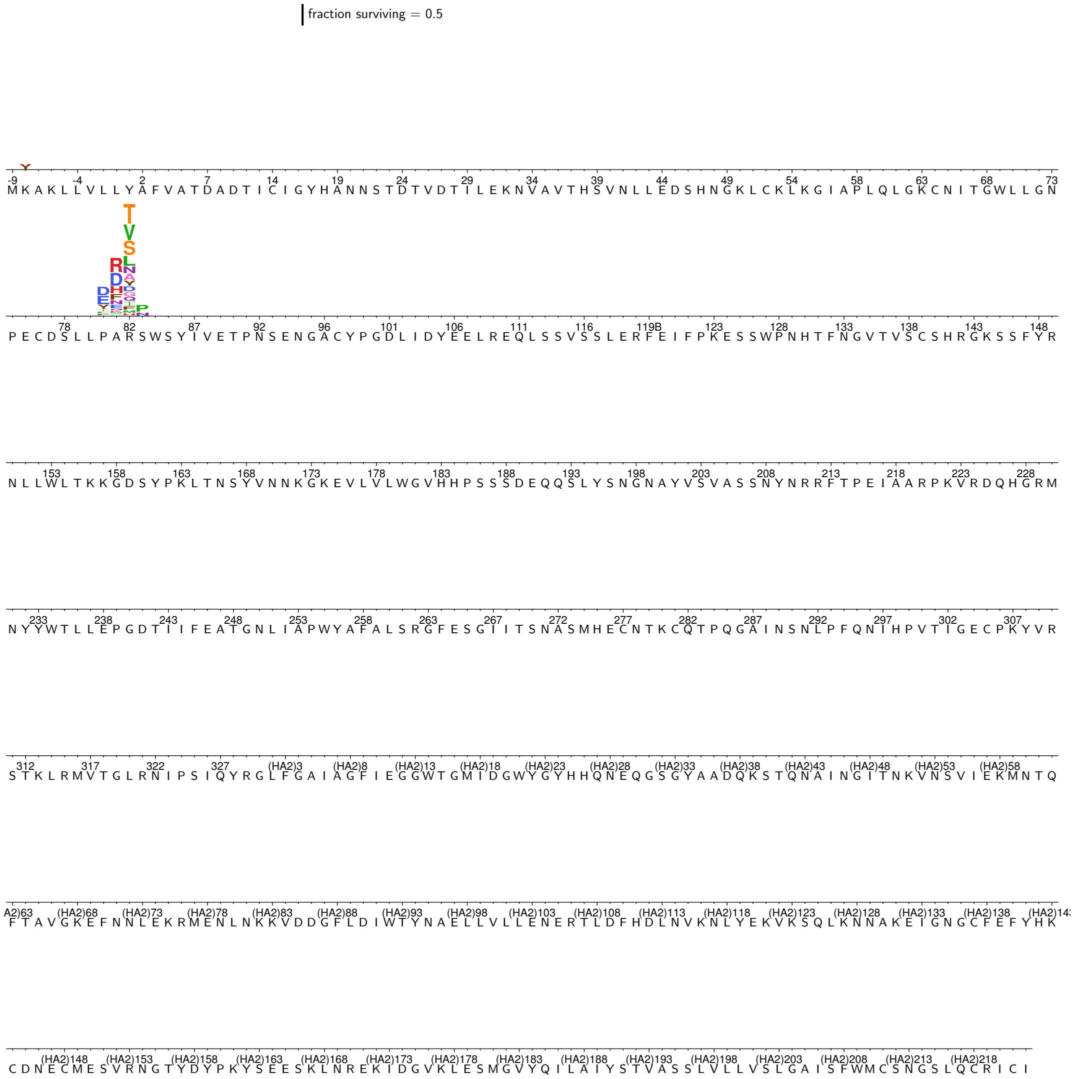

Supplementary Fig. 5: The excess fraction surviving selection with antibody H17L7 for all amino-acid mutations. The excess fraction surviving for each replicate was computed using Equation 2, then we took the median across all technical and biological replicates for each antibody concentration, and then took the medians of those values across concentrations. The height of each letter is proportional to the excess fraction surviving of virions with that mutation. The scale bar at the top of the plot relates the letter heights to the actual fractions. The sites are labeled using H3 numbering.

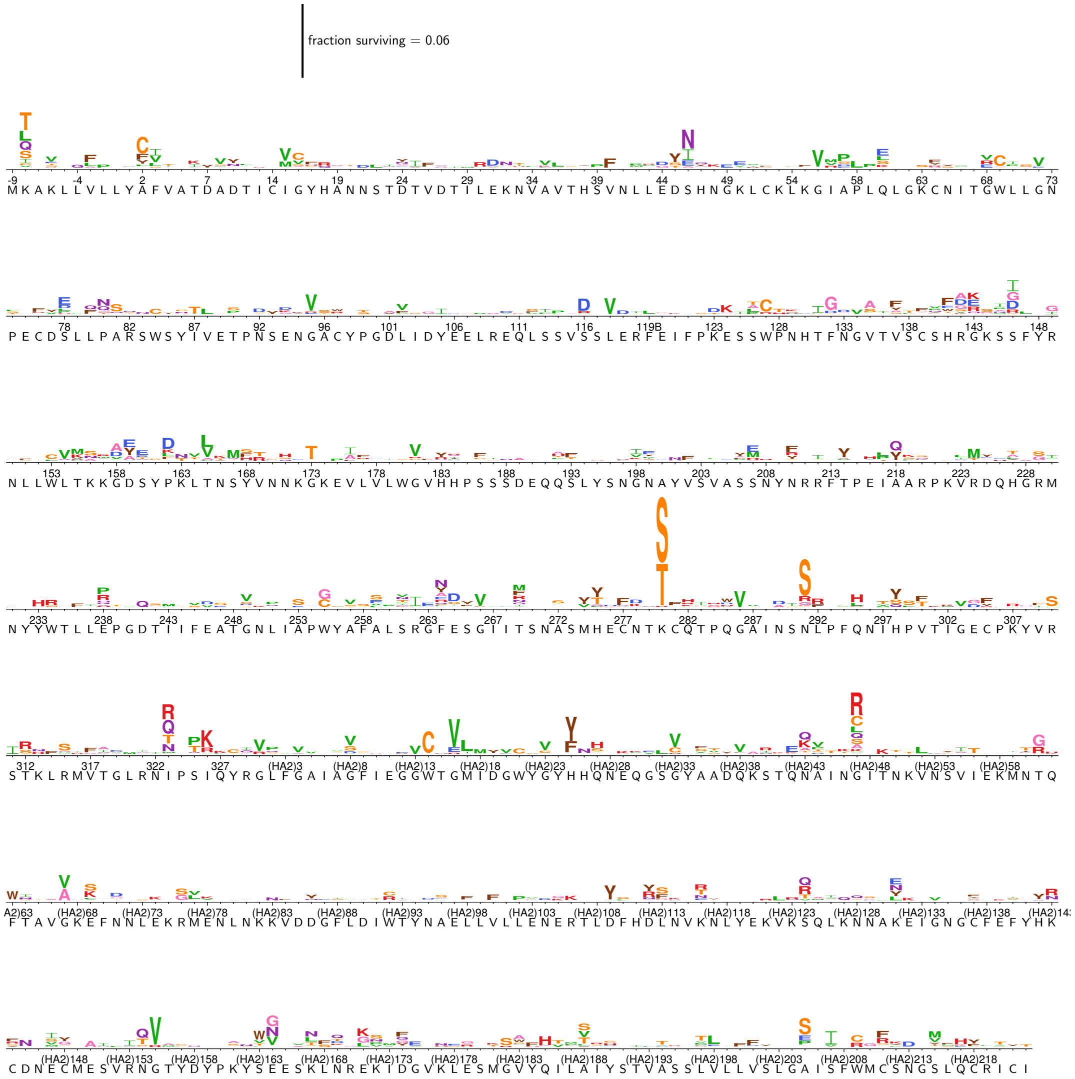

Supplementary Fig. 6: The excess fraction surviving selection with antibody FI6v3 for all amino-acid mutations. The excess fraction surviving for each replicate was computed using Equation 2, then we took the median across all technical and biological replicates for each antibody concentration, and then took the medians of those values across concentrations. The height of each letter is proportional to the excess fraction surviving of virions with that mutation. The scale bar at the top of the plot relates the letter heights to the actual fractions. The sites are labeled using H3 numbering.

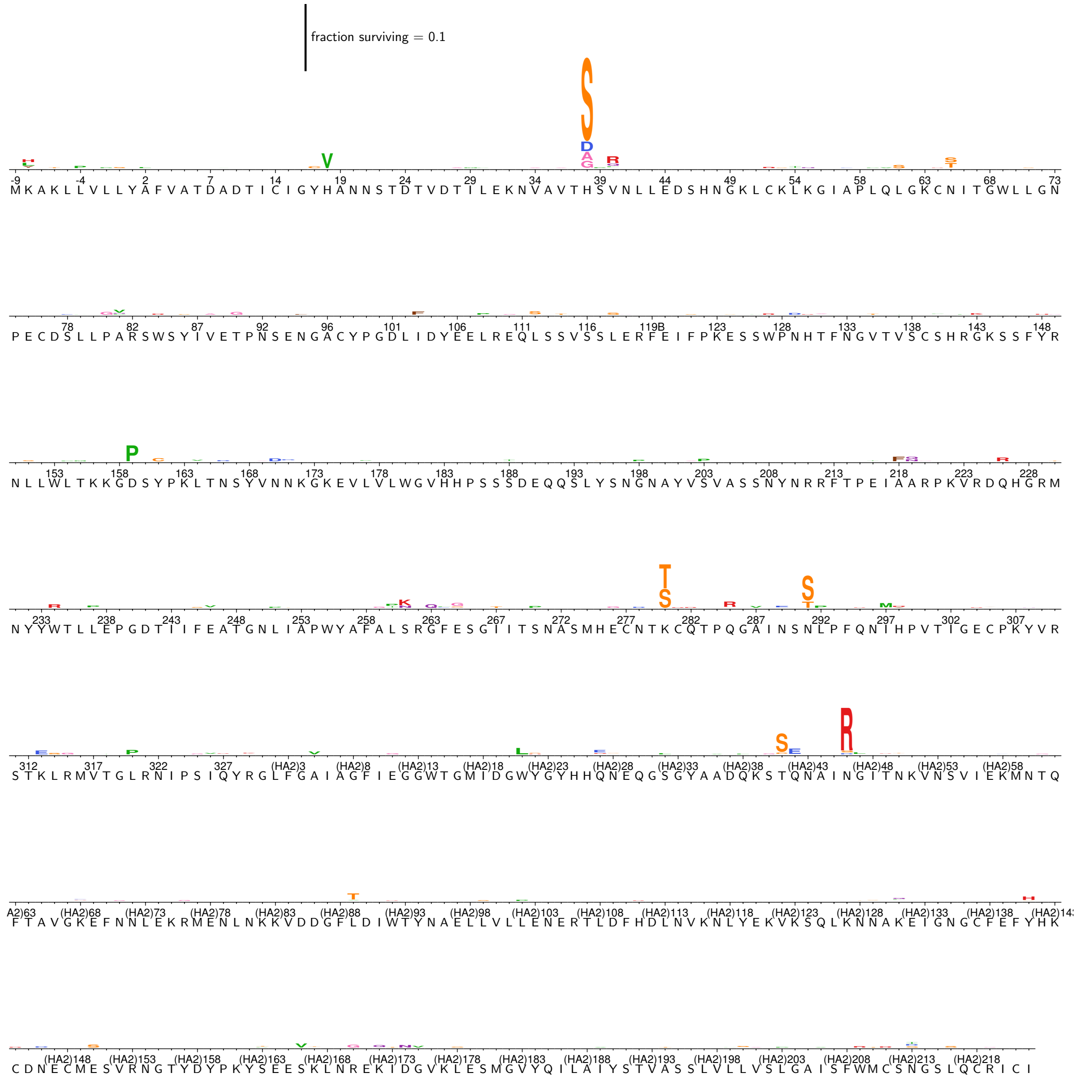

Supplementary Fig. 7: The excess fraction surviving selection with antibody C179 for all amino-acid mutations. The excess fraction surviving for each replicate was computed using Equation 2, then we took the median across all technical and biological replicates for each antibody concentration, and then took the medians of those values across concentrations. The height of each letter is proportional to the excess fraction surviving of virions with that mutation. The scale bar at the top of the plot relates the letter heights to the actual fractions. The sites are labeled using H3 numbering.

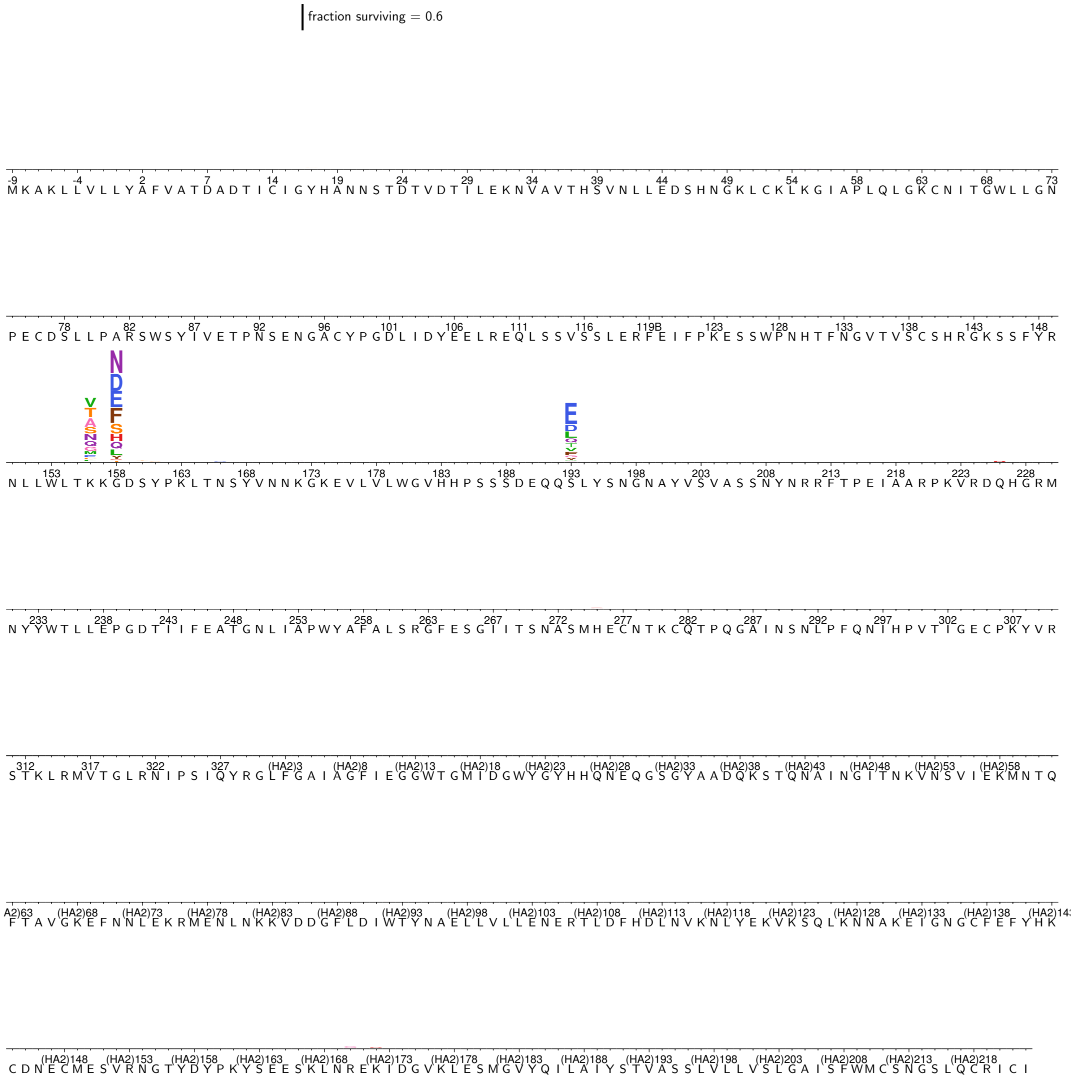

Supplementary Fig. 8: The excess fraction surviving selection with antibody S139/1 for all amino-acid mutations. The excess fraction surviving for each replicate was computed using Equation 2, then we took the median across all technical and biological replicates for each antibody concentration, and then took the medians of those values across concentrations. The height of each letter is proportional to the excess fraction surviving of virions with that mutation. The scale bar at the top of the plot relates the letter heights to the actual fractions. The sites are labeled using H3 numbering.

A

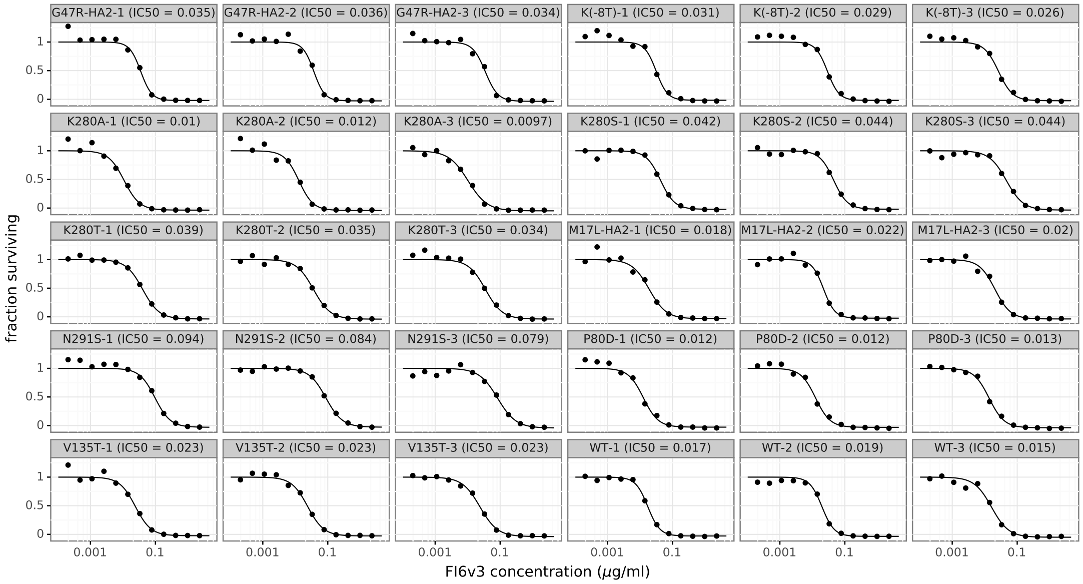

B

variant	replicate-1	replicate-2	replicate-3	mean	Pcorr
K280S	0.042	0.044	0.044	0.043	0.00072
G47R-HA2	0.035	0.036	0.034	0.035	0.0037
K280T	0.039	0.035	0.034	0.036	0.0087
N291S	0.094	0.084	0.079	0.086	0.023
K(-8T)	0.031	0.029	0.026	0.029	0.04
K280A	0.01	0.012	0.0097	0.011	0.12
V135T	0.023	0.023	0.023	0.023	0.27
P80D	0.012	0.012	0.013	0.013	0.42
M17L-HA2	0.018	0.022	0.02	0.02	1
WT	0.017	0.019	0.015	0.017	NaN

Supplementary Fig. 9: Replicates of the FI6v3 neutralization curves in Figure 6A. The neutralization assays were performed in triplicate for all nine mutants and wildtype. Figure 6A shows the *average* of those replicates. (A) The neutralization data for each replicate shown individually, with IC50 values fit using a four-parameter logistic curve with the top value constrained to one (see `https://jbloomlab.github.io/dms_tools2/dms_tools2.neutcurve.html` for the code used for the fitting.) (B) Table of the IC50 values for each replicate. We used an unpaired Student's t-test with unequal variances to test the null hypothesis that each mutant had an IC50 indistinguishable from wildtype. We then used Bonferroni's method to correct the *P*-values for multiple testing, and report these corrected values.

antibody	concentration (μg/ml)	replicate	fraction surviving
FI6v3	0.1	1a	0.01662
FI6v3	0.1	1b	0.01390
FI6v3	0.2	1a	0.00465
FI6v3	0.2	1b	0.00345
FI6v3	0.1	2	0.02322
FI6v3	0.2	2	0.00278
FI6v3	0.1	3	0.00903
FI6v3	0.2	3	0.00144
S139/1	100.0	1	0.02490
S139/1	200.0	1	0.01470
S139/1	300.0	1	0.01270
S139/1	100.0	2	0.02190
S139/1	200.0	2	0.01720
S139/1	300.0	2	0.00854
S139/1	100.0	3	0.05180
S139/1	200.0	3	0.04060
S139/1	300.0	3	0.03750
C179	1.0	1a	0.00941
C179	1.0	1b	0.00890
C179	1.0	1c	0.00960
C179	2.5	1	0.00450
C179	1.0	2	0.00554
C179	2.5	2	0.00198
C179	1.0	3	0.00256
C179	2.5	3	0.00100

Supplementary Table 1: The total fraction of virions surviving each antibody treatment at each concentration as estimated by qPCR. These are the quantities referred to as γ. This table shows the values for the broad antibodies; values for the narrow H17-L17, H17-L10, and H17-L7 antibodies have been reported previously[47].
