# Supplementary material for: How single mutations affect viral escape from broad and narrow antibodies to H1 influenza hemagglutinin
Source: Nat Commun. 2018 Apr 11;9:1386. doi: 10.1038/s41467-018-03665-3 (PMC5895760; doi:10.1038/s41467-018-03665-3)
Supplement: Supplementary file 2 — Description of Additional Supplementary Files(PDF 77 kb) [file 41467_2018_3665_MOESM2_ESM.pdf]

Description of Additional Supplementary Files

File Name: **Supplementary Data 1**
Description: **Conversion from sequential numbering of the A/WSN/1933 HA to H3 numbering.** In this CSV file, the *original* column gives the residue number in sequential (1, 2, ...) numbering of the A/WSN/1933 HA, and the *new* column gives the residue number in H3 numbering.

File Name: **Supplementary Data 2**
Description: **Sequences used to infer the tree for all HA subtypes.** This FASTA file gives the HA sequences used to infer the tree of subtypes in Figure 2.

File Name: **Supplementary Data 3**
Description: **Computer code and data for the analysis of the mutational anti-genic profiling data.** The code in this ZIP file performs the entire computational analysis beginning with downloading the FASTQ files from the Sequence Read Archive. The ZIP file contains a `README` file that explains the contents in detail. The actual analysis is performed by the Jupyter notebook `analysis_notebook.ipynb`, which includes embedded plots summarizing key statistics and results. An HTML version of this notebook is also included as Supplementary Data 4.

File Name: **Supplementary Data 4**
Description: **HTML version of the analysis notebook.** This file is an HTML rendering of the Jupyter notebook in Supplementary Data 3. It contains detailed plots for all aspects of the deep sequencing data and its analysis.

File Name: **Supplementary Data 5**
Description: **The excess fraction surviving for each mutation for each antibody.** This file is a ZIP of CSV files giving the numerical values plotted in the logo plots. These are median excess fraction surviving taken first across replicates and then across antibody concentrations. See Equation 2.

File Name: **Supplementary Data 6**
Description: **The fraction surviving for each mutation for each antibody.** This file differs from Supplementary Data 5 only in that the values are *not* adjusted to be in excess of the library average (e.g., they are from Equation 1 rather than Equation 2).
