# Supplementary material for: How single mutations affect viral escape from broad and narrow antibodies to H1 influenza hemagglutinin
Source: Nat Commun. 2018 Apr 11;9:1386. doi: 10.1038/s41467-018-03665-3 (PMC5895760; doi:10.1038/s41467-018-03665-3)
Supplement: Supplementary file 6 — Supplementary Data 4 [file 41467_2018_3665_MOESM6_ESM.html]

analysis\_notebook


# Table of Contents

- Ease of escape from broad and narrow antibodies against influenza HA
  - Overview
  - Experimental summary
- Import modules, define directories
- Define the samples
- Download `FASTQ` files from the SRA
- Align the deep sequencing data and count mutations
- Renumber codon counts to H3 numbering
- Quantifying the ease of escape from different antibodies
  - Make logoplots on the same scale
  - Make logoplot of WSN HA preferences
- Copy key pieces of data to paper directory

# Ease of escape from broad and narrow antibodies against influenza HA¶

Experiments and data analysis by Mike Doud, Juhye Lee, and Jesse Bloom.
The analysis uses the dms\_tools2 software package.

## Overview¶

We performed mutational antigenic profiling of three broadly neutralizing monoclonal antibodies, **FI6v3**, **S139/1**, and **C179**, that target influenza hemagglutinin. We used mutant virus libraries of the A/WSN/1933 (H1N1) HA using the procedure described in Doud, Hensley, and Bloom (2017). We are also including analysis of the monoclonal antibodies **H17-L19**, **H17-L10**, and **H17-L7**, previously profiled in Doud, Hensley, and Bloom (2017).

We then analyzed the data both to determine the antigenic effects of all mutations with respect to each antibody, and to compare the overall ease of escape across antibodies.

## Experimental summary¶

We selected three biological replicate mutant virus libraries carrying mutant HA's with different neutralizing concentrations of FI6v3, S139/1, C179, or one of the three H17-L\* antibodies (performed previously). We then infected cells with the neutralized viruses, extracted RNA from the infected cells, reverse-transcribed and amplified the extracted RNA, and deep sequenced the libraries using a barcoded-subamplicon approach.

In this analysis, we examine deep sequencing data from these antibody selections and evaluate amino-acid mutations that enable escape from each of these six antibodies.

# Import modules, define directories¶

In [1]:

```
import os
import glob
import math
import time
import pandas
import numpy
from IPython.display import display, HTML
import dms_tools2
import dms_tools2.plot
import dms_tools2.sra
import dms_tools2.diffsel
import dms_tools2.fracsurvive
from dms_tools2.ipython_utils import showPDF

print('Using dms_tools2 version {0}'.format(dms_tools2.__version__))

# define created directories
resultsdir = './results/'
if not os.path.isdir(resultsdir):
    os.mkdir(resultsdir)
    
fastqdir = os.path.join(resultsdir, 'FASTQ_files/')
if not os.path.isdir(fastqdir):
    os.mkdir(fastqdir)

countsdir = os.path.join(resultsdir, 'codoncounts/')
if not os.path.isdir(countsdir):
    os.mkdir(countsdir)
    
renumberedcountsdir = os.path.join(resultsdir, 'renumberedcounts')
if not os.path.isdir(renumberedcountsdir):
    os.mkdir(renumberedcountsdir)

fracsurvivedir = os.path.join(resultsdir, 'fracsurvive/')
if not os.path.isdir(fracsurvivedir):
    os.mkdir(fracsurvivedir)
    
fracsurviveaboveavgdir = os.path.join(resultsdir, 'fracsurviveaboveavg/')
if not os.path.isdir(fracsurviveaboveavgdir):
    os.mkdir(fracsurviveaboveavgdir)
    
prefsdir = os.path.join(resultsdir, 'prefs')
if not os.path.isdir(prefsdir):
    os.mkdir(prefsdir)
    
# CPUs to use, should not exceed the number you request with slurm
ncpus = 4 

# do we use existing results or generate everything new?
use_existing = 'yes'
```

```
/Users/jbloom/Library/Python/3.6/lib/python/site-packages/statsmodels/compat/pandas.py:56: FutureWarning: The pandas.core.datetools module is deprecated and will be removed in a future version. Please use the pandas.tseries module instead.
  from pandas.core import datetools
```

```
Using dms_tools2 version 2.2.1
```

# Define the samples¶

As input data, we have a CSV file (data/samples.csv) that defines the samples.
We read this file into a pandas dataframe that lists the sample names with the following associated information:

1. SRA run number
2. the antibody used for selection (`none` for mock selections and WT plasmid control)
3. the concentration (ug/ml) of antibody used to neutralize the libraries
4. the fraction infectivity after neutralization, as estimated by qPCR

In [2]:

```
samples = pandas.read_csv('./data/samples.csv')
display(HTML(samples.to_html(index=False)))
```

| name | run | antibody | ug/ml\_Ab | libfracsurvive |
| --- | --- | --- | --- | --- |
| L1-FI6v3-100ng-ml-r1 | SRR5930379 | FI6v3 | 0.1 | 0.01662 |
| L1-FI6v3-100ng-ml-r2 | SRR5930376 | FI6v3 | 0.1 | 0.01390 |
| L1-FI6v3-200ng-ml-r1 | SRR5930378 | FI6v3 | 0.2 | 0.00465 |
| L1-FI6v3-200ng-ml-r2 | SRR5930383 | FI6v3 | 0.2 | 0.00345 |
| L2-FI6v3-100ng-ml | SRR5930381 | FI6v3 | 0.1 | 0.02322 |
| L2-FI6v3-200ng-ml | SRR5930380 | FI6v3 | 0.2 | 0.00278 |
| L3-FI6v3-100ng-ml | SRR5930384 | FI6v3 | 0.1 | 0.00903 |
| L3-FI6v3-200ng-ml | SRR5930375 | FI6v3 | 0.2 | 0.00144 |
| L1-S139-100ug-ml | SRR6116992 | S139 | 100.0 | 0.02490 |
| L1-S139-200ug-ml | SRR6116993 | S139 | 200.0 | 0.01470 |
| L1-S139-300ug-ml | SRR6116994 | S139 | 300.0 | 0.01270 |
| L2-S139-100ug-ml | SRR6116990 | S139 | 100.0 | 0.02190 |
| L2-S139-200ug-ml | SRR6116995 | S139 | 200.0 | 0.01720 |
| L2-S139-300ug-ml | SRR6116996 | S139 | 300.0 | 0.00854 |
| L3-S139-100ug-ml | SRR6117015 | S139 | 100.0 | 0.05180 |
| L3-S139-200ug-ml | SRR6117012 | S139 | 200.0 | 0.04060 |
| L3-S139-300ug-ml | SRR6117013 | S139 | 300.0 | 0.03750 |
| L1-C179-1ug-ml-r1 | SRR6116997 | C179 | 1.0 | 0.00941 |
| L1-C179-1ug-ml-r2 | SRR6117006 | C179 | 1.0 | 0.00890 |
| L1-C179-1ug-ml-r3 | SRR6117007 | C179 | 1.0 | 0.00960 |
| L1-C179-2ug-ml | SRR6116998 | C179 | 2.5 | 0.00450 |
| L2-C179-1ug-ml | SRR6117010 | C179 | 1.0 | 0.00554 |
| L2-C179-2ug-ml | SRR6117011 | C179 | 2.5 | 0.00198 |
| L3-C179-1ug-ml | SRR6117016 | C179 | 1.0 | 0.00256 |
| L3-C179-2ug-ml | SRR6117017 | C179 | 2.5 | 0.00100 |
| L1-H17L19-1ug-ml-r1 | SRR4841570 | H17L19 | 1.0 | 0.00740 |
| L1-H17L19-1ug-ml-r2 | SRR4841573 | H17L19 | 1.0 | 0.00646 |
| L1-H17L19-10ug-ml-r1 | SRR4841567 | H17L19 | 10.0 | 0.00295 |
| L1-H17L19-10ug-ml-r2 | SRR4841574 | H17L19 | 10.0 | 0.00231 |
| L2-H17L19-1ug-ml | SRR4841572 | H17L19 | 1.0 | 0.01849 |
| L2-H17L19-10ug-ml | SRR4841575 | H17L19 | 10.0 | 0.00437 |
| L3-H17L19-1ug-ml | SRR4841595 | H17L19 | 1.0 | 0.01809 |
| L3-H17L19-10ug-ml | SRR4841596 | H17L19 | 10.0 | 0.00434 |
| L1-H17L10-3ug-ml | SRR4841597 | H17L10 | 3.0 | 0.00189 |
| L2-H17L10-3ug-ml | SRR4841598 | H17L10 | 3.0 | 0.00169 |
| L3-H17L10-3ug-ml | SRR4841599 | H17L10 | 3.0 | 0.00149 |
| L1-H17L7-15ug-ml | SRR4841600 | H17L7 | 15.0 | 0.00151 |
| L2-H17L7-15ug-ml | SRR4841601 | H17L7 | 15.0 | 0.00119 |
| L3-H17L7-15ug-ml | SRR4841602 | H17L7 | 15.0 | 0.00124 |
| L1-mock-r1-A | SRR4841578 | none | 0.0 | 1.00000 |
| L1-mock-r2-A | SRR4841581 | none | 0.0 | 1.00000 |
| L2-mock-A | SRR4841580 | none | 0.0 | 1.00000 |
| L3-mock-A | SRR4841582 | none | 0.0 | 1.00000 |
| L1-mock-r1-B | SRR6117000 | none | 0.0 | 1.00000 |
| L1-mock-r2-B | SRR6117001 | none | 0.0 | 1.00000 |
| L2-mock-B | SRR6117002 | none | 0.0 | 1.00000 |
| L3-mock-B | SRR6117003 | none | 0.0 | 1.00000 |
| WTplasmid | SRR6116999 | none | 0.0 | 1.00000 |

# Download `FASTQ` files from the SRA¶

All of the FASTQ files are on the Sequence Read Archive (SRA) under the run numbers listed in the `samples` dataframe defined above.
To download these files, we just pass that dataframe to the dms\_tools2.sra.fastqFromSRA function.
Note that this requires the `fastq-dump` and `aspera` programs to be installed on the computer you are using at the specified paths.

In [3]:

```
print('Downloading FASTQ files from the SRA...')
dms_tools2.sra.fastqFromSRA(
        samples=samples,
        fastq_dump='fastq-dump', # valid path to this program on the Hutch server
        fastqdir=fastqdir,
        aspera=(
            '/app/aspera-connect/3.5.1/bin/ascp', # valid path to ascp on Hutch server
            '/app/aspera-connect/3.5.1/etc/asperaweb_id_dsa.openssh' # Aspera key on Hutch server
            ),
        overwrite={'no':True, 'yes':False}[use_existing],
        )
print('Completed download of FASTQ files from the SRA')
```

```
Downloading FASTQ files from the SRA...
Completed download of FASTQ files from the SRA
```

# Align the deep sequencing data and count mutations¶

We used a barcoded-subamplicon sequencing approach to prep and deep sequence the libraries to high accuracy, as described in Doud, Hensley, and Bloom (2017).
We will now align the deep sequencing reads using dms2\_batch\_bcsubamp.

It is also worth noting that we have specified trimming parameters for Read 1 and Read 2.
Because different sets of samples were sequenced on different Illumina runs, these trimming parameters also differ across the samples.
The trimming was designed to clip off the worst-quality regions of each read for each sample.

We align to the reference sequence in data/WSN\_HA\_reference.fa.

In [4]:

```
# file containing wildtype WSN HA sequence
refseq = './data/WSN_HA_reference.fa'

# define subamplicon alignment specifications
alignspecs = ' '.join(['1,285,36,37', 
                       '286,570,31,32',
                       '571,855,37,32',
                       '856,1140,31,36',
                       '1141,1425,29,33',
                       '1426,1698,40,43'])

# Define trimming parameters
# All S139-selected samples have subamplicon-specific trimming parameters
# The other samples have trimming parameters of 200 for R1trim and 170 for R2trim
R1trim = 200
R2trim = 170
S139_R1trim = ' '.join(['190', '175', '175', '175', '190', '190'])
S139_R2trim = ' '.join(['180', '180', '180', '180', '190', '190'])
samples = (samples
           .assign(R1trim=lambda x: 
                          numpy.where(x['name'].str.contains('S139'), S139_R1trim, R1trim),
                   R2trim=lambda x: 
                          numpy.where(x['name'].str.contains('S139'), S139_R2trim, R2trim)
                   )
           )

# write batch file for dms2_batch_bcsubamp
countsbatchfile = os.path.join(countsdir, 'batch.csv')
samples[['name', 'R1', 'R1trim', 'R2trim']].to_csv(countsbatchfile, index=False)

print('\nNow running dms2_batch_bcsubamp...')
log = !dms2_batch_bcsubamp \
        --batchfile {countsbatchfile} \
        --refseq {refseq} \
        --alignspecs {alignspecs} \
        --outdir {countsdir} \
        --summaryprefix summary \
        --fastqdir {fastqdir} \
        --ncpus {ncpus} \
        --use_existing {use_existing}  
print('Completed dms2_batch_bcsubamp.')
```

```
Now running dms2_batch_bcsubamp...
Completed dms2_batch_bcsubamp.
```

The summary plots from dms2\_batch\_bcsubamp will have this prefix since it is what was passed as `--summaryprefix`:

In [5]:

```
countsplotprefix = os.path.join(countsdir, 'summary')
```

This plot shows the number of reads per barcode:

In [6]:

```
showPDF(countsplotprefix + '_readstats.pdf')
```

This next plot shows the number of reads per barcode.
Overall read depth for the C179-associated samples is very good.
Some of the S139-selected samples look like they are oversequenced, while others look like they could have been sequenced to greater depth.
The two L1mock-A samples also look a little undersequenced.

In [7]:

```
showPDF(countsplotprefix + '_readsperbc.pdf')
```

In [8]:

```
showPDF(countsplotprefix + '_bcstats.pdf')
```

In [9]:

```
showPDF(countsplotprefix + '_depth.pdf')
```

In [10]:

```
showPDF(countsplotprefix + '_mutfreq.pdf')
```

In [11]:

```
showPDF(countsplotprefix + '_codonmuttypes.pdf')
```

In [12]:

```
showPDF(countsplotprefix + '_codonntchanges.pdf')
```

In [13]:

```
showPDF(countsplotprefix + '_singlentchanges.pdf')
```

Some of the FI6v3- and H17-L19- selected samples show signs of oxidative damage (enrichment of `G to T` and `C to A` mutations). In contrast, the C179- and S139/1- selected samples do not show much signs of oxidative damage. The two sets of mock-selected samples (A and B), which are actually two sequencing reactions of the same initial library prep, do show some signs of oxidative damage.

# Renumber codon counts to H3 numbering¶

We will now renumber the codon counts from sequential (1, 2, ... starting from the initial Met) to H3 numbering.
The signal peptide is numbered with negative values, and the HA1 and HA2 subunits are numbered separately.
This numbering scheme is based on an alignment to a PDB structure for an H3 HA 4HMG.
We will use:

1. The ./data/H1toH3\_renumber.csv file to convert the sequential numbers into the H3 numbering scheme
2. The dms\_tools2.utils.renumberSites function to renumber the codon counts files

The renumbered files will be created in a new `renumberedcounts` directory and will possess the same names as the original codon counts files created from dms2\_batch\_bcsubamp above.

In [14]:

```
renumberfile = './data/H1toH3_renumber.csv'

# counts files to renumber
countsfiles = glob.glob('{0}/*codoncounts.csv'.format(countsdir))

dms_tools2.utils.renumberSites(renumberfile, countsfiles, missing='drop', 
        outdir=renumberedcountsdir)
```

# Quantifying the ease of escape from different antibodies¶

We next use dms2\_batch\_fracsurvive to evaluate the ease of escape from different antibodies.
This analysis estimates the fraction surviving antibody treatment for virions with each mutation.
Importantly, this metric can be used to compare escape across different antibodies and different concentrations of antibodies.

We first define a `fracsurvivebatch` dataframe that we write to CSV format to use as input to dms2\_batch\_fracsurvive.
Note how this dataframe contains a `libfracsurvive` column that gives the fraction of the library remaining infectious after antibody selection.
This quantity was determined by qRT-PCR of RNA extracted from infected cells after library neutralization.

In [15]:

```
fracsurvivebatch = pandas.DataFrame.from_records([ 
        # H17L19 at 1 ug/ml
         ('H17L19-1ug-ml', 'replicate-1a', 'L1-H17L19-1ug-ml-r1', 'L1-mock-r1-A'), 
         ('H17L19-1ug-ml', 'replicate-1b', 'L1-H17L19-1ug-ml-r2', 'L1-mock-r2-A'),
         ('H17L19-1ug-ml', 'replicate-2', 'L2-H17L19-1ug-ml', 'L2-mock-A'), 
         ('H17L19-1ug-ml', 'replicate-3', 'L3-H17L19-1ug-ml', 'L3-mock-A'), 
         # H17L19 at 10 ug/ml
         ('H17L19-10ug-ml', 'replicate-1a', 'L1-H17L19-10ug-ml-r1', 'L1-mock-r1-A'),
         ('H17L19-10ug-ml', 'replicate-1b', 'L1-H17L19-10ug-ml-r2', 'L1-mock-r2-A'),
         ('H17L19-10ug-ml', 'replicate-2', 'L2-H17L19-10ug-ml', 'L2-mock-A'),
         ('H17L19-10ug-ml', 'replicate-3', 'L3-H17L19-10ug-ml', 'L3-mock-A'), 
         # H17L10 at 3 ug/ml
         ('H17L10-3ug-ml', 'replicate-1', 'L1-H17L10-3ug-ml', 'L1-mock-r1-A'), 
         ('H17L10-3ug-ml', 'replicate-2', 'L2-H17L10-3ug-ml', 'L2-mock-A'), 
         ('H17L10-3ug-ml', 'replicate-3', 'L3-H17L10-3ug-ml', 'L3-mock-A'), 
         # H17L7 at 15 ug/ml
         ('H17L7-15ug-ml', 'replicate-1', 'L1-H17L7-15ug-ml', 'L1-mock-r1-A'), 
         ('H17L7-15ug-ml', 'replicate-2', 'L2-H17L7-15ug-ml', 'L2-mock-A'), 
         ('H17L7-15ug-ml', 'replicate-3', 'L3-H17L7-15ug-ml', 'L3-mock-A'),
         # FI6v3 at 0.1 ug/ml (100 ng/ml)
         ('FI6v3-100ng-ml', 'replicate-1a', 'L1-FI6v3-100ng-ml-r1', 'L1-mock-r1-A'), 
         ('FI6v3-100ng-ml', 'replicate-1b', 'L1-FI6v3-100ng-ml-r2', 'L1-mock-r2-A'),
         ('FI6v3-100ng-ml', 'replicate-2', 'L2-FI6v3-100ng-ml', 'L2-mock-A'), 
         ('FI6v3-100ng-ml', 'replicate-3', 'L3-FI6v3-100ng-ml', 'L3-mock-A'), 
         # FI6v3 at 0.2 ug/ml (200 ng/ml)
         ('FI6v3-200ng-ml', 'replicate-1a', 'L1-FI6v3-200ng-ml-r1', 'L1-mock-r1-A'),
         ('FI6v3-200ng-ml', 'replicate-1b', 'L1-FI6v3-200ng-ml-r2', 'L1-mock-r2-A'), 
         ('FI6v3-200ng-ml', 'replicate-2', 'L2-FI6v3-200ng-ml', 'L2-mock-A'),
         ('FI6v3-200ng-ml', 'replicate-3', 'L3-FI6v3-200ng-ml', 'L3-mock-A'),
         # C179 at 1 ug/ml
         ('C179-1ug-ml', 'replicate-1a', 'L1-C179-1ug-ml-r1', 'L1-mock-r1-B'),
         ('C179-1ug-ml', 'replicate-1b', 'L1-C179-1ug-ml-r2', 'L1-mock-r1-B'),
         ('C179-1ug-ml', 'replicate-1c', 'L1-C179-1ug-ml-r3', 'L1-mock-r2-B'), 
         ('C179-1ug-ml', 'replicate-2', 'L2-C179-1ug-ml', 'L2-mock-B'), 
         ('C179-1ug-ml', 'replicate-3', 'L3-C179-1ug-ml', 'L3-mock-B'),
         # C179 at 2.5 ug/ml (rounded down to 2 ug/ml)
         ('C179-2ug-ml', 'replicate-1', 'L1-C179-2ug-ml', 'L1-mock-r1-B'), 
         ('C179-2ug-ml', 'replicate-2', 'L2-C179-2ug-ml', 'L2-mock-B'), 
         ('C179-2ug-ml', 'replicate-3', 'L3-C179-2ug-ml', 'L3-mock-B'), 
         # S139 at 100 ug/ml
         ('S139-100ug-ml', 'replicate-1', 'L1-S139-100ug-ml', 'L1-mock-r1-B'),  
         ('S139-100ug-ml', 'replicate-2', 'L2-S139-100ug-ml', 'L2-mock-B'),        
         ('S139-100ug-ml', 'replicate-3', 'L3-S139-100ug-ml', 'L3-mock-B'), 
         # S139 at 200 ug/ml
         ('S139-200ug-ml', 'replicate-1', 'L1-S139-200ug-ml', 'L1-mock-r1-B'),
         ('S139-200ug-ml', 'replicate-2', 'L2-S139-200ug-ml', 'L2-mock-B'),
         ('S139-200ug-ml', 'replicate-3', 'L3-S139-200ug-ml', 'L3-mock-B'), 
         # S139 at 300 ug/ml
         ('S139-300ug-ml', 'replicate-1', 'L1-S139-300ug-ml', 'L1-mock-r1-B'), 
         ('S139-300ug-ml', 'replicate-2', 'L2-S139-300ug-ml', 'L2-mock-B'),
         ('S139-300ug-ml', 'replicate-3', 'L3-S139-300ug-ml', 'L3-mock-B'), 
        ],       
        columns=['group', 'name', 'sel', 'mock']
        )

# all samples have the same error control
fracsurvivebatch['err'] = 'WTplasmid' 

# add the libfracsurvive values from the previously defined `samples` dataframe
fracsurvivebatch = pandas.merge(fracsurvivebatch, samples[['name', 'libfracsurvive']], 
        left_on = 'sel', right_on= 'name', suffixes=('', '_y')).drop('name_y', axis=1)

# make nicely formatted grouplabels (antibody names) for faceted plots
fracsurvivebatch['grouplabel'] = (fracsurvivebatch['group']
        .str.replace('-ml', '/ml)')
        .str.replace('-', ' (')
        .str.replace('ng', ' ng')
        .str.replace('ug', ' $\mu$g')
        .str.replace('S139', 'broad anti-RBS antibody: S139/1')
        .str.replace('H17', 'narrow anti-head antibody: H17')
        .str.replace('C179', 'broad anti-stalk antibody: C179')
        .str.replace('FI6v3', 'broad anti-stalk antibody: FI6v3')
        .str.replace('C179 \(2', 'C179 (2.5')
        )

# add column with antibody name, which is word item in group name
fracsurvivebatch['antibody'] = list(map(lambda g: g.split('-')[0], fracsurvivebatch['group']))

# display and write information
fracsurvivebatchfile = os.path.join(fracsurvivedir, 'batch.csv')
print("Here is the batch input that we write to the CSV file {0}:".format(fracsurvivebatchfile))
display(HTML(fracsurvivebatch.to_html(index=False)))
fracsurvivebatch.to_csv(fracsurvivebatchfile, index=False)
```

```
Here is the batch input that we write to the CSV file ./results/fracsurvive/batch.csv:
```

| group | name | sel | mock | err | libfracsurvive | grouplabel | antibody |
| --- | --- | --- | --- | --- | --- | --- | --- |
| H17L19-1ug-ml | replicate-1a | L1-H17L19-1ug-ml-r1 | L1-mock-r1-A | WTplasmid | 0.00740 | narrow anti-head antibody: H17L19 (1 $\mu$g/ml) | H17L19 |
| H17L19-1ug-ml | replicate-1b | L1-H17L19-1ug-ml-r2 | L1-mock-r2-A | WTplasmid | 0.00646 | narrow anti-head antibody: H17L19 (1 $\mu$g/ml) | H17L19 |
| H17L19-1ug-ml | replicate-2 | L2-H17L19-1ug-ml | L2-mock-A | WTplasmid | 0.01849 | narrow anti-head antibody: H17L19 (1 $\mu$g/ml) | H17L19 |
| H17L19-1ug-ml | replicate-3 | L3-H17L19-1ug-ml | L3-mock-A | WTplasmid | 0.01809 | narrow anti-head antibody: H17L19 (1 $\mu$g/ml) | H17L19 |
| H17L19-10ug-ml | replicate-1a | L1-H17L19-10ug-ml-r1 | L1-mock-r1-A | WTplasmid | 0.00295 | narrow anti-head antibody: H17L19 (10 $\mu$g/ml) | H17L19 |
| H17L19-10ug-ml | replicate-1b | L1-H17L19-10ug-ml-r2 | L1-mock-r2-A | WTplasmid | 0.00231 | narrow anti-head antibody: H17L19 (10 $\mu$g/ml) | H17L19 |
| H17L19-10ug-ml | replicate-2 | L2-H17L19-10ug-ml | L2-mock-A | WTplasmid | 0.00437 | narrow anti-head antibody: H17L19 (10 $\mu$g/ml) | H17L19 |
| H17L19-10ug-ml | replicate-3 | L3-H17L19-10ug-ml | L3-mock-A | WTplasmid | 0.00434 | narrow anti-head antibody: H17L19 (10 $\mu$g/ml) | H17L19 |
| H17L10-3ug-ml | replicate-1 | L1-H17L10-3ug-ml | L1-mock-r1-A | WTplasmid | 0.00189 | narrow anti-head antibody: H17L10 (3 $\mu$g/ml) | H17L10 |
| H17L10-3ug-ml | replicate-2 | L2-H17L10-3ug-ml | L2-mock-A | WTplasmid | 0.00169 | narrow anti-head antibody: H17L10 (3 $\mu$g/ml) | H17L10 |
| H17L10-3ug-ml | replicate-3 | L3-H17L10-3ug-ml | L3-mock-A | WTplasmid | 0.00149 | narrow anti-head antibody: H17L10 (3 $\mu$g/ml) | H17L10 |
| H17L7-15ug-ml | replicate-1 | L1-H17L7-15ug-ml | L1-mock-r1-A | WTplasmid | 0.00151 | narrow anti-head antibody: H17L7 (15 $\mu$g/ml) | H17L7 |
| H17L7-15ug-ml | replicate-2 | L2-H17L7-15ug-ml | L2-mock-A | WTplasmid | 0.00119 | narrow anti-head antibody: H17L7 (15 $\mu$g/ml) | H17L7 |
| H17L7-15ug-ml | replicate-3 | L3-H17L7-15ug-ml | L3-mock-A | WTplasmid | 0.00124 | narrow anti-head antibody: H17L7 (15 $\mu$g/ml) | H17L7 |
| FI6v3-100ng-ml | replicate-1a | L1-FI6v3-100ng-ml-r1 | L1-mock-r1-A | WTplasmid | 0.01662 | broad anti-stalk antibody: FI6v3 (100 ng/ml) | FI6v3 |
| FI6v3-100ng-ml | replicate-1b | L1-FI6v3-100ng-ml-r2 | L1-mock-r2-A | WTplasmid | 0.01390 | broad anti-stalk antibody: FI6v3 (100 ng/ml) | FI6v3 |
| FI6v3-100ng-ml | replicate-2 | L2-FI6v3-100ng-ml | L2-mock-A | WTplasmid | 0.02322 | broad anti-stalk antibody: FI6v3 (100 ng/ml) | FI6v3 |
| FI6v3-100ng-ml | replicate-3 | L3-FI6v3-100ng-ml | L3-mock-A | WTplasmid | 0.00903 | broad anti-stalk antibody: FI6v3 (100 ng/ml) | FI6v3 |
| FI6v3-200ng-ml | replicate-1a | L1-FI6v3-200ng-ml-r1 | L1-mock-r1-A | WTplasmid | 0.00465 | broad anti-stalk antibody: FI6v3 (200 ng/ml) | FI6v3 |
| FI6v3-200ng-ml | replicate-1b | L1-FI6v3-200ng-ml-r2 | L1-mock-r2-A | WTplasmid | 0.00345 | broad anti-stalk antibody: FI6v3 (200 ng/ml) | FI6v3 |
| FI6v3-200ng-ml | replicate-2 | L2-FI6v3-200ng-ml | L2-mock-A | WTplasmid | 0.00278 | broad anti-stalk antibody: FI6v3 (200 ng/ml) | FI6v3 |
| FI6v3-200ng-ml | replicate-3 | L3-FI6v3-200ng-ml | L3-mock-A | WTplasmid | 0.00144 | broad anti-stalk antibody: FI6v3 (200 ng/ml) | FI6v3 |
| C179-1ug-ml | replicate-1a | L1-C179-1ug-ml-r1 | L1-mock-r1-B | WTplasmid | 0.00941 | broad anti-stalk antibody: C179 (1 $\mu$g/ml) | C179 |
| C179-1ug-ml | replicate-1b | L1-C179-1ug-ml-r2 | L1-mock-r1-B | WTplasmid | 0.00890 | broad anti-stalk antibody: C179 (1 $\mu$g/ml) | C179 |
| C179-1ug-ml | replicate-1c | L1-C179-1ug-ml-r3 | L1-mock-r2-B | WTplasmid | 0.00960 | broad anti-stalk antibody: C179 (1 $\mu$g/ml) | C179 |
| C179-1ug-ml | replicate-2 | L2-C179-1ug-ml | L2-mock-B | WTplasmid | 0.00554 | broad anti-stalk antibody: C179 (1 $\mu$g/ml) | C179 |
| C179-1ug-ml | replicate-3 | L3-C179-1ug-ml | L3-mock-B | WTplasmid | 0.00256 | broad anti-stalk antibody: C179 (1 $\mu$g/ml) | C179 |
| C179-2ug-ml | replicate-1 | L1-C179-2ug-ml | L1-mock-r1-B | WTplasmid | 0.00450 | broad anti-stalk antibody: C179 (2.5 $\mu$g/ml) | C179 |
| C179-2ug-ml | replicate-2 | L2-C179-2ug-ml | L2-mock-B | WTplasmid | 0.00198 | broad anti-stalk antibody: C179 (2.5 $\mu$g/ml) | C179 |
| C179-2ug-ml | replicate-3 | L3-C179-2ug-ml | L3-mock-B | WTplasmid | 0.00100 | broad anti-stalk antibody: C179 (2.5 $\mu$g/ml) | C179 |
| S139-100ug-ml | replicate-1 | L1-S139-100ug-ml | L1-mock-r1-B | WTplasmid | 0.02490 | broad anti-RBS antibody: S139/1 (100 $\mu$g/ml) | S139 |
| S139-100ug-ml | replicate-2 | L2-S139-100ug-ml | L2-mock-B | WTplasmid | 0.02190 | broad anti-RBS antibody: S139/1 (100 $\mu$g/ml) | S139 |
| S139-100ug-ml | replicate-3 | L3-S139-100ug-ml | L3-mock-B | WTplasmid | 0.05180 | broad anti-RBS antibody: S139/1 (100 $\mu$g/ml) | S139 |
| S139-200ug-ml | replicate-1 | L1-S139-200ug-ml | L1-mock-r1-B | WTplasmid | 0.01470 | broad anti-RBS antibody: S139/1 (200 $\mu$g/ml) | S139 |
| S139-200ug-ml | replicate-2 | L2-S139-200ug-ml | L2-mock-B | WTplasmid | 0.01720 | broad anti-RBS antibody: S139/1 (200 $\mu$g/ml) | S139 |
| S139-200ug-ml | replicate-3 | L3-S139-200ug-ml | L3-mock-B | WTplasmid | 0.04060 | broad anti-RBS antibody: S139/1 (200 $\mu$g/ml) | S139 |
| S139-300ug-ml | replicate-1 | L1-S139-300ug-ml | L1-mock-r1-B | WTplasmid | 0.01270 | broad anti-RBS antibody: S139/1 (300 $\mu$g/ml) | S139 |
| S139-300ug-ml | replicate-2 | L2-S139-300ug-ml | L2-mock-B | WTplasmid | 0.00854 | broad anti-RBS antibody: S139/1 (300 $\mu$g/ml) | S139 |
| S139-300ug-ml | replicate-3 | L3-S139-300ug-ml | L3-mock-B | WTplasmid | 0.03750 | broad anti-RBS antibody: S139/1 (300 $\mu$g/ml) | S139 |

We now run dms2\_batch\_fracsurvive twice with the following difference:

1. First we run it simply computing the fraction surviving for each mutation.
2. Then we run it with the `--aboveavg yes` option to compute the fraction surviving for each mutation **above the overall library average**.

Note how the results for these two different runs are output to two different subdirectories.

In [16]:

```
for (arg_aboveavg, outdir) in [('', fracsurvivedir), 
                               ('--aboveavg yes', fracsurviveaboveavgdir)]:
    print("\nRunning dms2_batch_fracsurvive {0}and writing output to {1}".format(
            {'':'', '--aboveavg yes':'with `--aboveavg yes` '}[arg_aboveavg], outdir))
    log = !dms2_batch_fracsurvive \
            --summaryprefix summary \
            --batchfile {fracsurvivebatchfile} \
            --outdir {outdir} \
            --indir {renumberedcountsdir} \
            --use_existing {use_existing} \
            {arg_aboveavg} 
    print("Completed run.")
```

```
Running dms2_batch_fracsurvive and writing output to ./results/fracsurvive/
Completed run.

Running dms2_batch_fracsurvive with `--aboveavg yes` and writing output to ./results/fracsurviveaboveavg/
Completed run.
```

The results for the analysis with and without the `--aboveavg yes` option are extremely similar for the antibodies with strong escape mutations.
However, they are a bit clearer for the antibodies without strong escape mutations when we use the `--aboveavg yes` option.
So for all plots below, we will show the results for the analysis where we compute the average fraction surviving **above average**.
First, we define the prefix for the summary plots for the fraction surviving above average:

In [17]:

```
fracsurviveprefix = os.path.join(fracsurviveaboveavgdir, 'summary_')
```

First, we just look to see the correlations among the replicates for each antibody / concentration.
We do this looking at the *average fraction surviving* at each site, averaged over all mutations at that site.
These plots have the suffix `avgfracsurvive.corr.pdf`.
As seen below, the correlations are very good except for the broadly neutralizing anti-stalk antibodies FI6v3 and C179.
As we will see below, the correlations are worse for those antibodies as there are no strong escape mutants and so there is simply less signal to be seen.

In [18]:

```
for antibody in fracsurvivebatch['antibody'].unique():
    groups = fracsurvivebatch.query('antibody == @antibody')['group'].unique()
    plots = [fracsurviveprefix + g + '-avgfracsurvivecorr.pdf' for g in groups]
    print("\nReplicate correlations for {0}".format(antibody))
    showPDF(plots, width=300 * len(plots))
```

```
Replicate correlations for H17L19
```

```
Replicate correlations for H17L10
```

```
Replicate correlations for H17L7
```

```
Replicate correlations for FI6v3
```

```
Replicate correlations for C179
```

```
Replicate correlations for S139
```

Now we want to look across the HA sequence to see the fraction surviving above average for each antibody / concentration.
There are two ways that we can summarize replicates across an antibody / concentration: by taking the *mean*, or by taking the *median*.
Output plots have been created for both of these.
Here we use the *median*, as that looks slightly cleaner as it reduces the effects of outliers more.
However, the *mean* looks very similar (you can see this by looking the created plots that have `mean` in their name in place of `median`).

First we look at the fraction escape averaged across all mutations at a site:

In [19]:

```
showPDF(fracsurviveprefix + 'medianavgfracsurvive.pdf', width=800)
```

Now we look at the fraction escape for the amino-acid mutation with the maximum effect at each site:

In [20]:

```
showPDF(fracsurviveprefix + 'medianmaxfracsurvive.pdf', width=800)
```

When considering the plots above, a couple things are clear:

1. The mutation with the maximum fraction surviving has a much higher fraction surviving selections with any of the narrow anti-head antibodies or the broad S139/1 antibody than the broad anti-stalk antibodies C179 or FI6v3.
2. For all but the broad anti-stalk antibodies, the maximum fraction surviving reaches **very** high proportions (~70-80%!) for some mutants after selection with S139/1. This is as high or higher than what is seen for the narrow antibodies. But for the anti-stalk antibodies, no mutations have a high fractin surviving
3. Although S139/1, C179, and FI6v3 are all considered to be "broadly-neutralizing" against naturally occurring strains, there are clearly more good escape mutants to S139/1. This result suggests that breadth and ease of escape are not the same.
4. When comparing C179 vs FI6v3, a few mutations that confer escape from C179 reach higher fractions of escape than those fractions reached by escape mutations from FI6v3. Therefore, escape mutations from C179 seem have slightly higher effect sizes and have better "escape potential" than escape mutations from FI6v3.

Now for each antibody, we will take the median across antibody concentrations of the across-replicate medians, and use these to make logo plots for each antibody.

In [21]:

```
medianfiles = []
medavgsitefiles = []
logoplots = []
for antibody in fracsurvivebatch['antibody'].unique():
    print('\nGetting and plotting overall across-concentration median for {0}'.format(antibody))
    
    # list of files
    medianfracsurvive_files = glob.glob('{0}*{1}-*medianmutfracsurvive.csv'
            .format(fracsurviveprefix, antibody))
    
    # Average across mutation fraction surviving
    medianmutdf = dms_tools2.fracsurvive.avgMutFracSurvive(
            medianfracsurvive_files, 'median')
    
    # Convert median mutation to median avg site fraction surviving
    avgsitedf = dms_tools2.fracsurvive.mutToSiteFracSurvive(
            medianmutdf)
    
    # Write median mutation fracsurvive dataframe to csv files
    medianfile = os.path.join(fracsurviveaboveavgdir, 
            'antibody_{0}_median.csv'.format(antibody))
    medianfiles.append(medianfile)
    print("Writing across-concentration medians to {0}".format(
            medianfile))
    medianmutdf.to_csv(medianfile, index=False)
    
    # Write median average site fracsurvive dataframe to csv files
    avgsitefile = os.path.join(fracsurviveaboveavgdir,
            'antibody_{0}_median_avgsite.csv'.format(antibody))
    medavgsitefiles.append(avgsitefile)
    print("Writing across-concentration site medians to {0}".format(
            avgsitefile))
    avgsitedf.to_csv(avgsitefile, index=False)
    
    # now make logo plot
    # scale bar unit is maximum effect
    scaleunit = '{0:.1g}'.format(medianmutdf['mutfracsurvive'].max())
    scalelabel = '"fraction surviving = {0}"'.format(scaleunit)
    logoplot = os.path.join(fracsurviveaboveavgdir,
            '{0}_fracsurvive.pdf'.format(antibody))
    logoplots.append(logoplot)
    print("Creating logo plot {0} for {1} from {2}".format(
            logoplot, antibody, medianfile))
    log = !dms2_logoplot \
            --fracsurvive {medianfile} \
            --name {antibody} \
            --outdir {fracsurviveaboveavgdir} \
            --numberevery 5 \
            --nperline 81 \
            --underlay yes \
            --overlay1 {medianfile} wildtype wildtype \
            --scalebar {scaleunit} {scalelabel} \
            --use_existing {use_existing}

    showPDF(logoplot)
```

```
Getting and plotting overall across-concentration median for H17L19
Writing across-concentration medians to ./results/fracsurviveaboveavg/antibody_H17L19_median.csv
Writing across-concentration site medians to ./results/fracsurviveaboveavg/antibody_H17L19_median_avgsite.csv
Creating logo plot ./results/fracsurviveaboveavg/H17L19_fracsurvive.pdf for H17L19 from ./results/fracsurviveaboveavg/antibody_H17L19_median.csv
```

```
Getting and plotting overall across-concentration median for H17L10
Writing across-concentration medians to ./results/fracsurviveaboveavg/antibody_H17L10_median.csv
Writing across-concentration site medians to ./results/fracsurviveaboveavg/antibody_H17L10_median_avgsite.csv
Creating logo plot ./results/fracsurviveaboveavg/H17L10_fracsurvive.pdf for H17L10 from ./results/fracsurviveaboveavg/antibody_H17L10_median.csv
```

```
Getting and plotting overall across-concentration median for H17L7
Writing across-concentration medians to ./results/fracsurviveaboveavg/antibody_H17L7_median.csv
Writing across-concentration site medians to ./results/fracsurviveaboveavg/antibody_H17L7_median_avgsite.csv
Creating logo plot ./results/fracsurviveaboveavg/H17L7_fracsurvive.pdf for H17L7 from ./results/fracsurviveaboveavg/antibody_H17L7_median.csv
```

```
Getting and plotting overall across-concentration median for FI6v3
Writing across-concentration medians to ./results/fracsurviveaboveavg/antibody_FI6v3_median.csv
Writing across-concentration site medians to ./results/fracsurviveaboveavg/antibody_FI6v3_median_avgsite.csv
Creating logo plot ./results/fracsurviveaboveavg/FI6v3_fracsurvive.pdf for FI6v3 from ./results/fracsurviveaboveavg/antibody_FI6v3_median.csv
```

```
Getting and plotting overall across-concentration median for C179
Writing across-concentration medians to ./results/fracsurviveaboveavg/antibody_C179_median.csv
Writing across-concentration site medians to ./results/fracsurviveaboveavg/antibody_C179_median_avgsite.csv
Creating logo plot ./results/fracsurviveaboveavg/C179_fracsurvive.pdf for C179 from ./results/fracsurviveaboveavg/antibody_C179_median.csv
```

```
Getting and plotting overall across-concentration median for S139
Writing across-concentration medians to ./results/fracsurviveaboveavg/antibody_S139_median.csv
Writing across-concentration site medians to ./results/fracsurviveaboveavg/antibody_S139_median_avgsite.csv
Creating logo plot ./results/fracsurviveaboveavg/S139_fracsurvive.pdf for S139 from ./results/fracsurviveaboveavg/antibody_S139_median.csv
```

Now we just repeat the above but do it using the fraction surviving values that are **not** corrected to be above average.
We don't show these plots in the notebook, we just create them.

In [22]:

```
fracsurviveprefix_notexcess = os.path.join(fracsurvivedir, 'summary_')
medianfiles_notexcess = []
medavgsitefiles_notexcess = []
for antibody in fracsurvivebatch['antibody'].unique():
    print('\nGetting and plotting overall across-concentration median for {0}'.format(antibody))
    
    # list of files
    medianfracsurvive_files = glob.glob('{0}*{1}-*medianmutfracsurvive.csv'
            .format(fracsurviveprefix_notexcess, antibody))
    
    # Average across mutation fraction surviving
    medianmutdf = dms_tools2.fracsurvive.avgMutFracSurvive(
            medianfracsurvive_files, 'median')
    
    # Convert median mutation to median avg site fraction surviving
    avgsitedf = dms_tools2.fracsurvive.mutToSiteFracSurvive(
            medianmutdf)
    
    # Write median mutation fracsurvive dataframe to csv files
    medianfile = os.path.join(fracsurvivedir, 
            'antibody_{0}_median.csv'.format(antibody))
    medianfiles_notexcess.append(medianfile)
    print("Writing across-concentration medians to {0}".format(
            medianfile))
    medianmutdf.to_csv(medianfile, index=False)
    
    # Write median average site fracsurvive dataframe to csv files
    avgsitefile = os.path.join(fracsurvivedir,
            'antibody_{0}_median_avgsite.csv'.format(antibody))
    medavgsitefiles_notexcess.append(avgsitefile)
    print("Writing across-concentration site medians to {0}".format(
            avgsitefile))
    avgsitedf.to_csv(avgsitefile, index=False)
    
    # now make logo plot
    # scale bar unit is maximum effect
    scaleunit = '{0:.1g}'.format(medianmutdf['mutfracsurvive'].max())
    scalelabel = '"fraction surviving = {0}"'.format(scaleunit)
    logoplot = os.path.join(fracsurvivedir,
            '{0}_fracsurvive.pdf'.format(antibody))
    print("Creating logo plot {0} for {1} from {2}".format(
            logoplot, antibody, medianfile))
    log = !dms2_logoplot \
            --fracsurvive {medianfile} \
            --name {antibody} \
            --outdir {fracsurvivedir} \
            --numberevery 5 \
            --nperline 81 \
            --underlay yes \
            --overlay1 {medianfile} wildtype wildtype \
            --scalebar {scaleunit} {scalelabel} \
            --use_existing {use_existing}
```

```
Getting and plotting overall across-concentration median for H17L19
Writing across-concentration medians to ./results/fracsurvive/antibody_H17L19_median.csv
Writing across-concentration site medians to ./results/fracsurvive/antibody_H17L19_median_avgsite.csv
Creating logo plot ./results/fracsurvive/H17L19_fracsurvive.pdf for H17L19 from ./results/fracsurvive/antibody_H17L19_median.csv

Getting and plotting overall across-concentration median for H17L10
Writing across-concentration medians to ./results/fracsurvive/antibody_H17L10_median.csv
Writing across-concentration site medians to ./results/fracsurvive/antibody_H17L10_median_avgsite.csv
Creating logo plot ./results/fracsurvive/H17L10_fracsurvive.pdf for H17L10 from ./results/fracsurvive/antibody_H17L10_median.csv

Getting and plotting overall across-concentration median for H17L7
Writing across-concentration medians to ./results/fracsurvive/antibody_H17L7_median.csv
Writing across-concentration site medians to ./results/fracsurvive/antibody_H17L7_median_avgsite.csv
Creating logo plot ./results/fracsurvive/H17L7_fracsurvive.pdf for H17L7 from ./results/fracsurvive/antibody_H17L7_median.csv

Getting and plotting overall across-concentration median for FI6v3
Writing across-concentration medians to ./results/fracsurvive/antibody_FI6v3_median.csv
Writing across-concentration site medians to ./results/fracsurvive/antibody_FI6v3_median_avgsite.csv
Creating logo plot ./results/fracsurvive/FI6v3_fracsurvive.pdf for FI6v3 from ./results/fracsurvive/antibody_FI6v3_median.csv

Getting and plotting overall across-concentration median for C179
Writing across-concentration medians to ./results/fracsurvive/antibody_C179_median.csv
Writing across-concentration site medians to ./results/fracsurvive/antibody_C179_median_avgsite.csv
Creating logo plot ./results/fracsurvive/C179_fracsurvive.pdf for C179 from ./results/fracsurvive/antibody_C179_median.csv

Getting and plotting overall across-concentration median for S139
Writing across-concentration medians to ./results/fracsurvive/antibody_S139_median.csv
Writing across-concentration site medians to ./results/fracsurvive/antibody_S139_median_avgsite.csv
Creating logo plot ./results/fracsurvive/S139_fracsurvive.pdf for S139 from ./results/fracsurvive/antibody_S139_median.csv
```

## Make logoplots on the same scale¶

The logoplots above are each dynamically scaled according to the range of the data.
For the broad antibodies, we also want to make logoplots that share the same scale.
These will be used for some of the figures.

Specifically, we make logo plots for FI6v3 and C179 that share a maximum, and one for S139/1 that has 10 times that maximum.

In [23]:

```
fracsurvivemax = 0.4 # for C179 / FI6v3
scaleheight = 0.1 # scale bar height for C179 / FI6v3
s139factor = 10 # max for S139/1 this much larger

for f in medianfiles:
    if ('C179' in f) or ('FI6v3' in f):
        abmax = fracsurvivemax
        abscaleheight = scaleheight
    elif 'S139' in f:
        abmax = s139factor * fracsurvivemax
        abscaleheight = s139factor * abscaleheight
    else:
        continue
        
    antibody = f.split('_')[1]
    abname = antibody + '-scaled'
    logoplot = os.path.join(fracsurviveaboveavgdir, '{0}_fracsurvive.pdf'.format(abname))
    scalelabel = '"fraction surviving = {0:.1f}"'.format(abscaleheight)
    
    print("Creating logo plot {0} for {1} from {2}".format(logoplot, antibody, f))
    log = !dms2_logoplot \
            --fracsurvive {f} \
            --name {abname} \
            --outdir {fracsurviveaboveavgdir} \
            --numberevery 5 \
            --nperline 81 \
            --underlay yes \
            --overlay1 {f} wildtype wildtype \
            --fracsurvivemax {abmax} \
            --scalebar {abscaleheight} {scalelabel} \
            --use_existing no
```

```
Creating logo plot ./results/fracsurviveaboveavg/FI6v3-scaled_fracsurvive.pdf for FI6v3 from ./results/fracsurviveaboveavg/antibody_FI6v3_median.csv
Creating logo plot ./results/fracsurviveaboveavg/C179-scaled_fracsurvive.pdf for C179 from ./results/fracsurviveaboveavg/antibody_C179_median.csv
Creating logo plot ./results/fracsurviveaboveavg/S139-scaled_fracsurvive.pdf for S139 from ./results/fracsurviveaboveavg/antibody_S139_median.csv
```

## Make logoplot of WSN HA preferences¶

We want to see how the sites of escape mutations relate to the inherent mutational tolerance of HA.
This inherent mutations tolerance was measured by Doud and Bloom (2016), who determined the preference of each site in HA for each possible amino acid.
We will make a logoplot visualizing these preferences.
We will use the **rescaled** preferences averaged across all replicates; these preferences are in H3 numbering.
These preferences are taken from the original publication, and are in the file ./data/Overall-WSNHA\_merged\_prefs\_rescaled\_H3numbering.csv.

In [24]:

```
# Define preferences file
prefsfile = './data/Overall-WSNHA_merged_prefs_rescaled_H3numbering.csv'

logoname = 'WSNprefs-H3numbering'

log = !dms2_logoplot \
        --prefs {prefsfile} \
        --name {logoname} \
        --outdir {prefsdir} \
        --nperline 81 \
        --use_existing no
        
logoplot = os.path.join(prefsdir, '{0}_prefs.pdf'.format(logoname))
showPDF(logoplot)
```

# Copy key pieces of data to paper directory¶

We copy key files / data to the paper figure directory.

In [30]:

```
figsdir = '../paper/figs/'
!cp {fracsurviveprefix}medianavgfracsurvive.pdf {figsdir}/avgfracsurvive.pdf
!cp {fracsurviveprefix}medianmaxfracsurvive.pdf {figsdir}/maxfracsurvive.pdf
!cp {' '.join(logoplots)} {figsdir}/logoplots/
!cp {' '.join(medianfiles)} {figsdir}/medianfracsurvivefiles_excess/
!cp {' '.join(medavgsitefiles)} {figsdir}/medianfracsurvivefiles_excess/
!cp {' '.join(medianfiles_notexcess)} {figsdir}/medianfracsurvivefiles/
!cp {' '.join(medavgsitefiles_notexcess)} {figsdir}/medianfracsurvivefiles/
!cp {fracsurviveprefix}*avgfracsurvivecorr.pdf {figsdir}/corrs/
```
