# Supplementary material for: How single mutations affect viral escape from broad and narrow antibodies to H1 influenza hemagglutinin
Source: Nat Commun. 2018 Apr 11;9:1386. doi: 10.1038/s41467-018-03665-3 (PMC5895760; doi:10.1038/s41467-018-03665-3)
Supplement: Supplementary file 5 — Supplementary Data 3(ZIP 19513 kb) [file 41467_2018_3665_MOESM5_ESM.zip › analysis_code/results/codoncounts/summary_codonmuttypes.pdf]

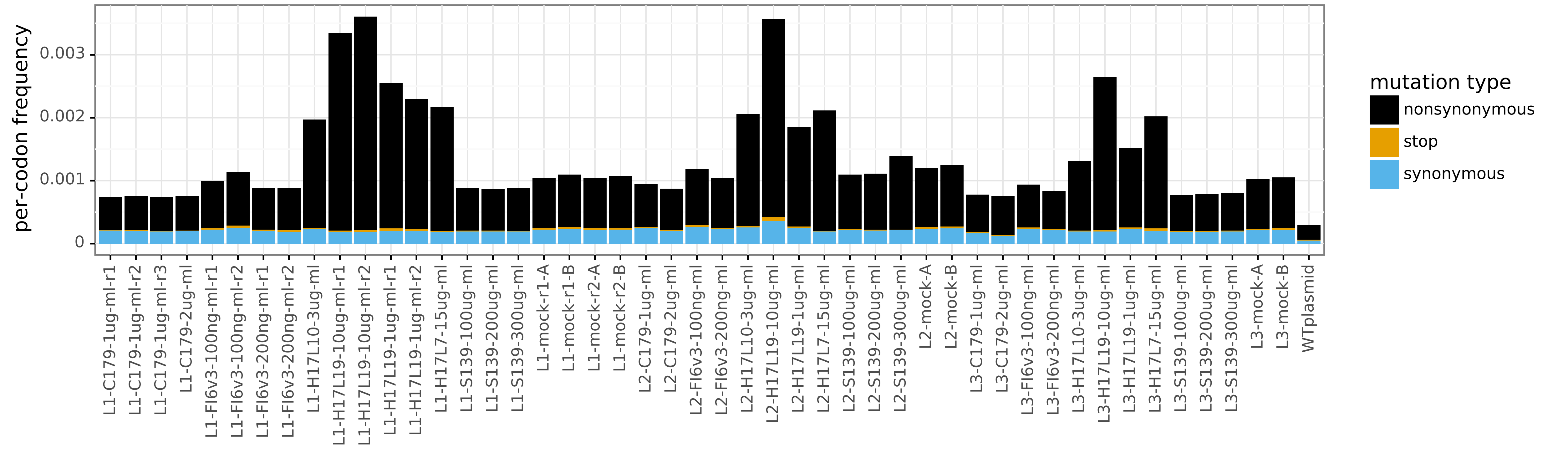
per-codon frequency
0.003
0.002
0.001
0
L1-C179-1ug-ml-r1
L1-C179-1ug-ml-r2
L1-C179-1ug-ml-r3
L1-C179-2ug-ml
L1-FI6v3-100ng-ml-r1
L1-FI6v3-100ng-ml-r2
L1-FI6v3-200ng-ml-r1
L1-FI6v3-200ng-ml-r2
L1-H17L10-3ug-ml
L1-H17L19-10ug-ml-r1
L1-H17L19-10ug-ml-r2
L1-H17L19-1ug-ml-r1
L1-H17L19-1ug-ml-r2
L1-H17L7-15ug-ml
L1-S139-100ug-ml
L1-S139-200ug-ml
L1-S139-300ug-ml
L1-mock-r1-A
L1-mock-r1-B
L1-mock-r2-A
L1-mock-r2-B
L2-C179-1ug-ml
L2-C179-2ug-ml
L2-FI6v3-100ng-ml
L2-FI6v3-200ng-ml
L2-H17L10-3ug-ml
L2-H17L19-10ug-ml
L2-H17L19-1ug-ml
L2-H17L7-15ug-ml
L2-S139-100ug-ml
L2-S139-200ug-ml
L2-S139-300ug-ml
L2-mock-A
L2-mock-B
L3-C179-1ug-ml
L3-C179-2ug-ml
L3-FI6v3-100ng-ml
L3-FI6v3-200ng-ml
L3-H17L10-3ug-ml
L3-H17L19-10ug-ml
L3-H17L19-1ug-ml
L3-H17L7-15ug-ml
L3-S139-100ug-ml
L3-S139-200ug-ml
L3-S139-300ug-ml
L3-mock-A
L3-mock-B
WTplasmid
mutation type
nonsynonymous
stop
synonymous
