# Supplementary material for: How single mutations affect viral escape from broad and narrow antibodies to H1 influenza hemagglutinin
Source: Nat Commun. 2018 Apr 11;9:1386. doi: 10.1038/s41467-018-03665-3 (PMC5895760; doi:10.1038/s41467-018-03665-3)
Supplement: Supplementary file 5 — Supplementary Data 3(ZIP 19513 kb) [file 41467_2018_3665_MOESM5_ESM.zip › analysis_code/results/fracsurvive/summary_C179-1ug-ml-maxfracsurvivecorr.pdf]

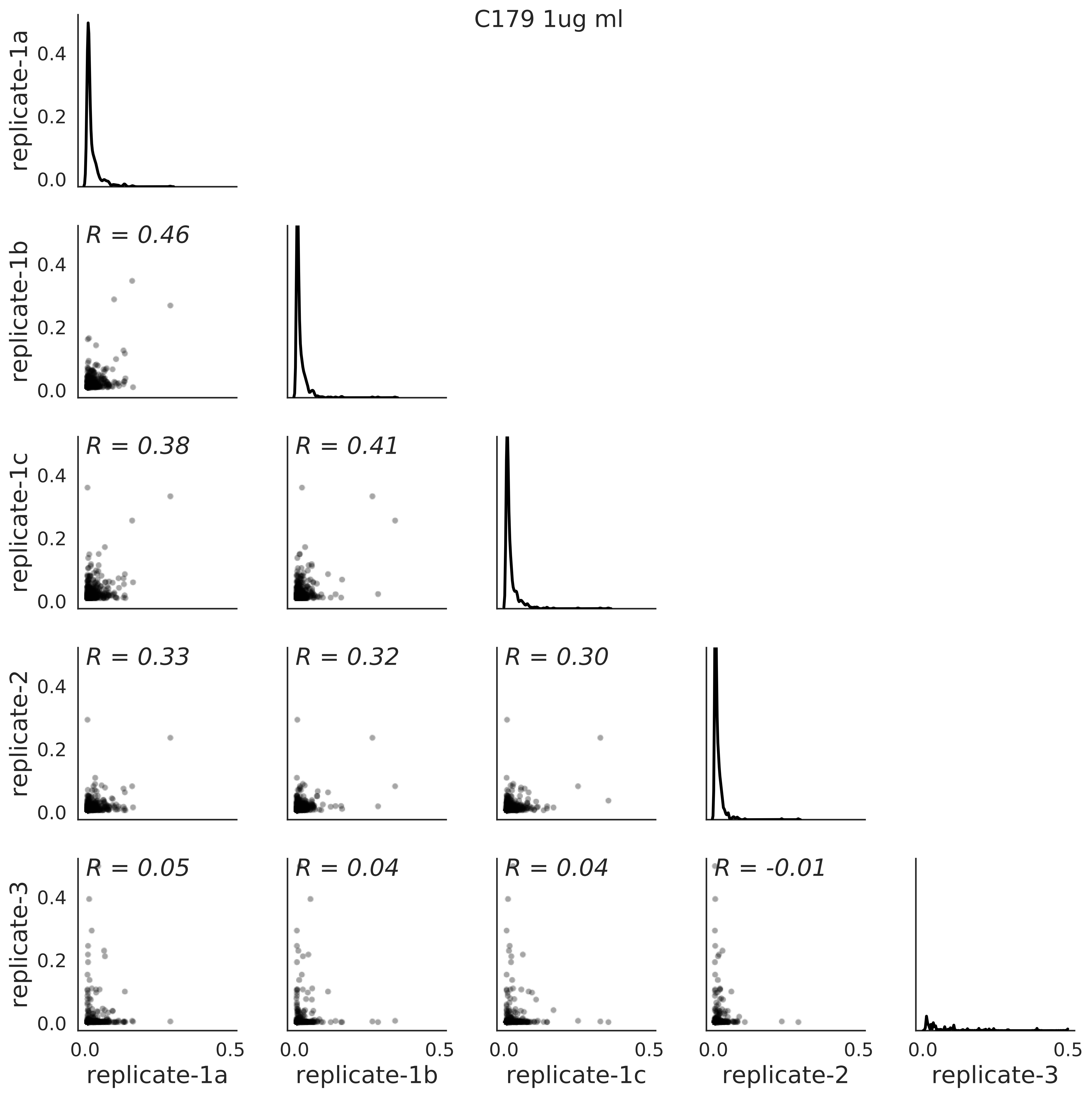
C179 1ug ml
replicate-1a
replicate-1b
replicate-1c
replicate-2
replicate-3
R = 0.46
R = 0.38
R = 0.41
R = 0.33
R = 0.32
R = 0.30
R = 0.05
R = 0.04
R = 0.04
R = -0.01
0.0
0.2
0.4
0.5
