# Supplementary material for: How single mutations affect viral escape from broad and narrow antibodies to H1 influenza hemagglutinin
Source: Nat Commun. 2018 Apr 11;9:1386. doi: 10.1038/s41467-018-03665-3 (PMC5895760; doi:10.1038/s41467-018-03665-3)
Supplement: Supplementary file 5 — Supplementary Data 3(ZIP 19513 kb) [file 41467_2018_3665_MOESM5_ESM.zip › analysis_code/results/fracsurvive/summary_H17L7-15ug-ml-mutfracsurvivecorr.pdf]

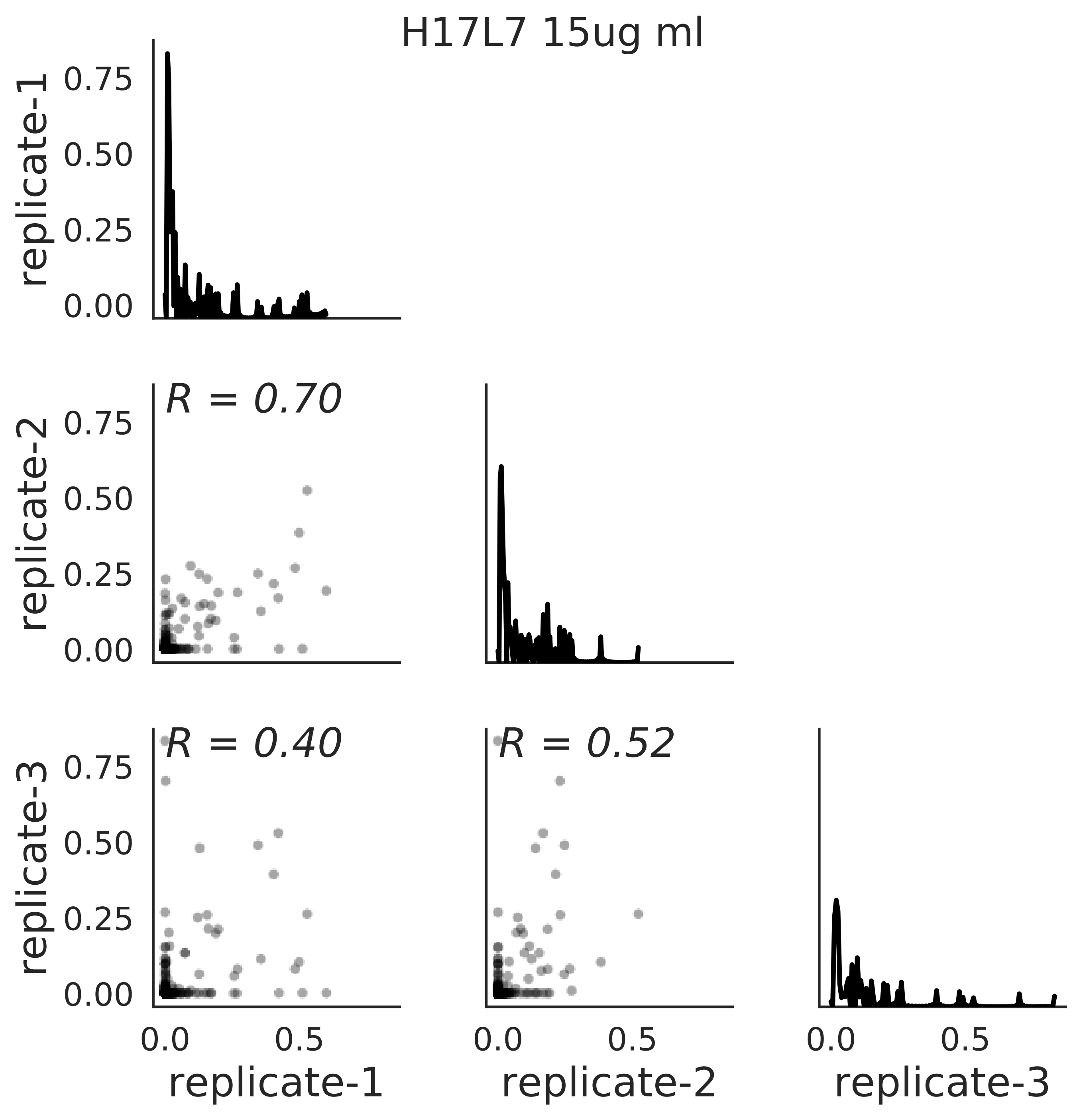
H17L7 15ug ml
replicate-1
replicate-2
replicate-3
R = 0.70
R = 0.40
R = 0.52
0.00
0.25
0.50
0.75
0.0
0.5
replicate-1
replicate-2
replicate-3
