# Supplementary material for: How single mutations affect viral escape from broad and narrow antibodies to H1 influenza hemagglutinin
Source: Nat Commun. 2018 Apr 11;9:1386. doi: 10.1038/s41467-018-03665-3 (PMC5895760; doi:10.1038/s41467-018-03665-3)
Supplement: Supplementary file 5 — Supplementary Data 3(ZIP 19513 kb) [file 41467_2018_3665_MOESM5_ESM.zip › analysis_code/results/fracsurviveaboveavg/C179-scaled_fracsurvive.pdf]

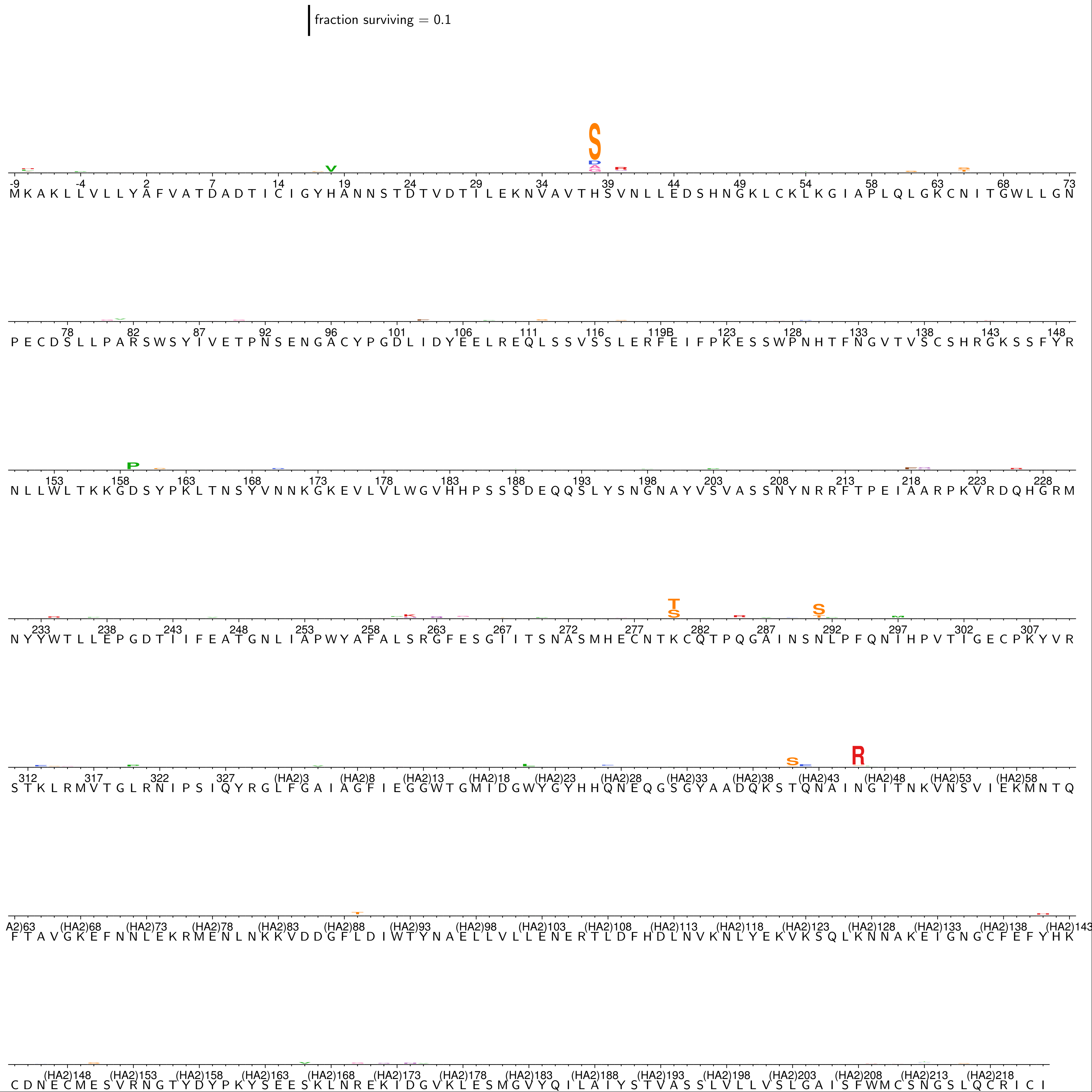
fraction surviving = 0.1
-9 -4 2 7 14 19 24 29 34 39 44 49 54 58 63 68 73
M K A K L L V L L Y A F V A T D A D T I C I G Y H A N N S T D T V D T I L E K N V A V T H S V N L L E D S H N G K L C K L K G I A P L Q L G K C N I T G W L L G N
78 82 87 92 96 101 106 111 116 119B 123 128 133 138 143 148
P E C D S L L P A R S W S Y I V E T P N S E N G A C Y P G D L I D Y E E L R E Q L S S V S S L E R F E I F P K E S S W P N H T F N G V T V S C S H R G K S S F Y R
153 158 163 168 173 178 183 188 193 198 203 208 213 218 223 228
N L L W L T K K G D S Y P K L T N S Y V N N K G K E V L V L W G V H H P S S S D E Q Q S L Y S N G N A Y V S V A S S N Y N R R F T P E I A A R P K V R D Q H G R M
233 238 243 248 253 258 263 267 272 277 282 287 292 297 302 307
N Y Y W T L L E P G D T I I F E A T G N L I A P W Y A F A L S R G F E S G I I T S N A S M H E C N T K C Q T P Q G A I N S N L P F Q N I H P V T I G E C P K Y V R
312 317 322 327 (HA2)3 (HA2)8 (HA2)13 (HA2)18 (HA2)23 (HA2)28 (HA2)33 (HA2)38 (HA2)43 (HA2)48 (HA2)53 (HA2)58
S T K L R M V T G L R N I P S I Q Y R G L F G A I A G F I E G G W T G M I D G W Y G Y H H Q N E Q G S G Y A A D Q K S T Q N A I N G I T N K V N S V I E K M N T Q
A2)63 (HA2)68 (HA2)73 (HA2)78 (HA2)83 (HA2)88 (HA2)93 (HA2)98 (HA2)103 (HA2)108 (HA2)113 (HA2)118 (HA2)123 (HA2)128 (HA2)133 (HA2)138 (HA2)143
F T A V G K E F N N L E K R M E N L N K K V D D G F L D I W T Y N A E L L V L L E N E R T L D F H D L N V K N L Y E K V K S Q L K N N A K E I G N G C F E F Y H K
(HA2)148 (HA2)153 (HA2)158 (HA2)163 (HA2)168 (HA2)173 (HA2)178 (HA2)183 (HA2)188 (HA2)193 (HA2)198 (HA2)203 (HA2)208 (HA2)213 (HA2)218
C D N E C M E S V R N G T Y D Y P K Y S E E S K L N R E K I D G V K L E S M G V Y Q I L A I Y S T V A S S L V L L V S L G A I S F W M C S N G S L Q C R I C I
