# Supplementary material for: How single mutations affect viral escape from broad and narrow antibodies to H1 influenza hemagglutinin
Source: Nat Commun. 2018 Apr 11;9:1386. doi: 10.1038/s41467-018-03665-3 (PMC5895760; doi:10.1038/s41467-018-03665-3)
Supplement: Supplementary file 5 — Supplementary Data 3(ZIP 19513 kb) [file 41467_2018_3665_MOESM5_ESM.zip › analysis_code/results/fracsurviveaboveavg/summary_FI6v3-100ng-ml-avgfracsurvivecorr.pdf]

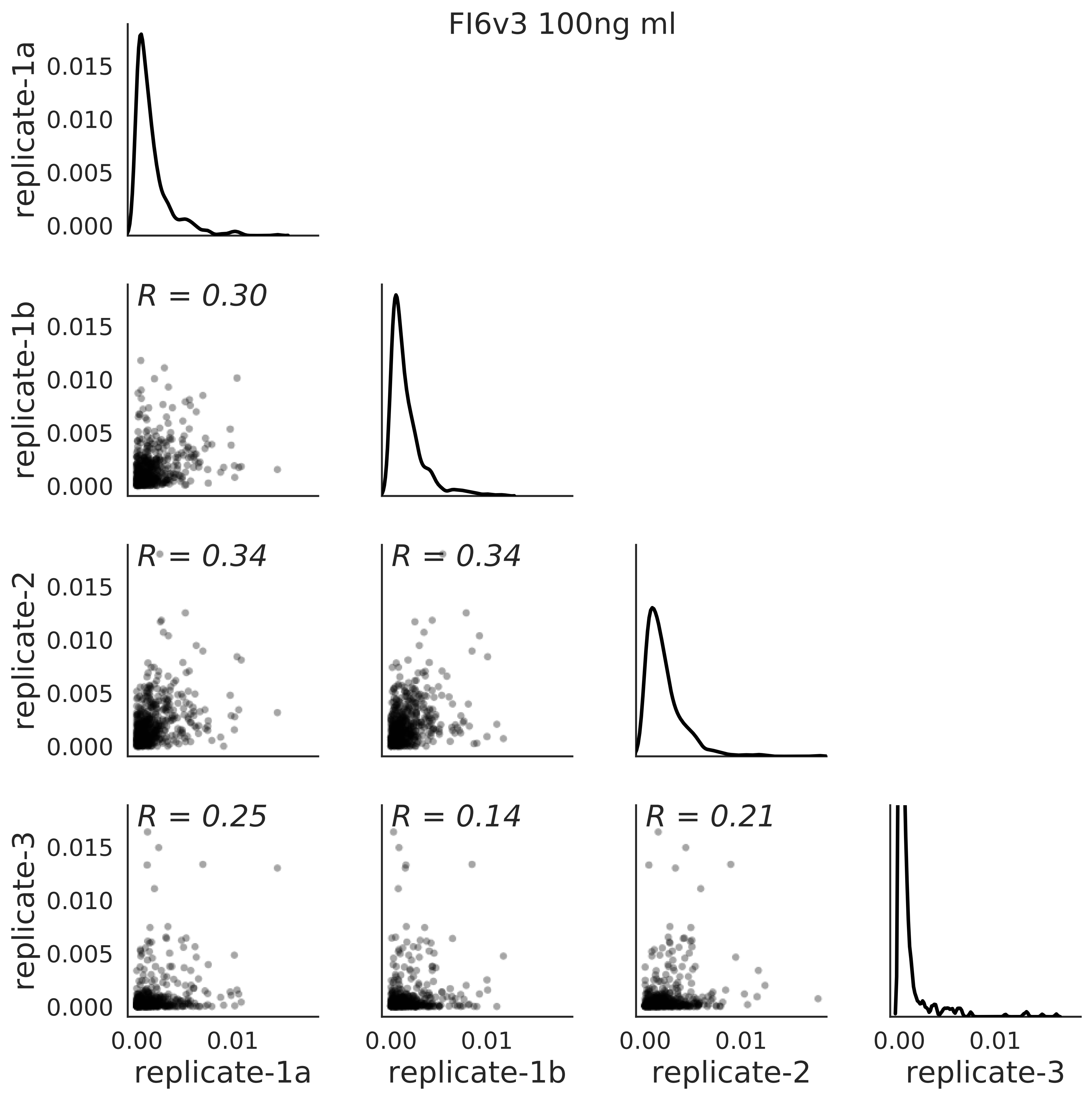
FI6v3 100ng ml
replicate-1a
replicate-1b
replicate-2
replicate-3
R = 0.30
R = 0.34
R = 0.34
R = 0.25
R = 0.14
R = 0.21
0.000
0.005
0.010
0.015
0.00
0.01
