# Supplementary material for: How single mutations affect viral escape from broad and narrow antibodies to H1 influenza hemagglutinin
Source: Nat Commun. 2018 Apr 11;9:1386. doi: 10.1038/s41467-018-03665-3 (PMC5895760; doi:10.1038/s41467-018-03665-3)
Supplement: Supplementary file 5 — Supplementary Data 3(ZIP 19513 kb) [file 41467_2018_3665_MOESM5_ESM.zip › analysis_code/results/fracsurviveaboveavg/summary_H17L19-1ug-ml-avgfracsurvivecorr.pdf]

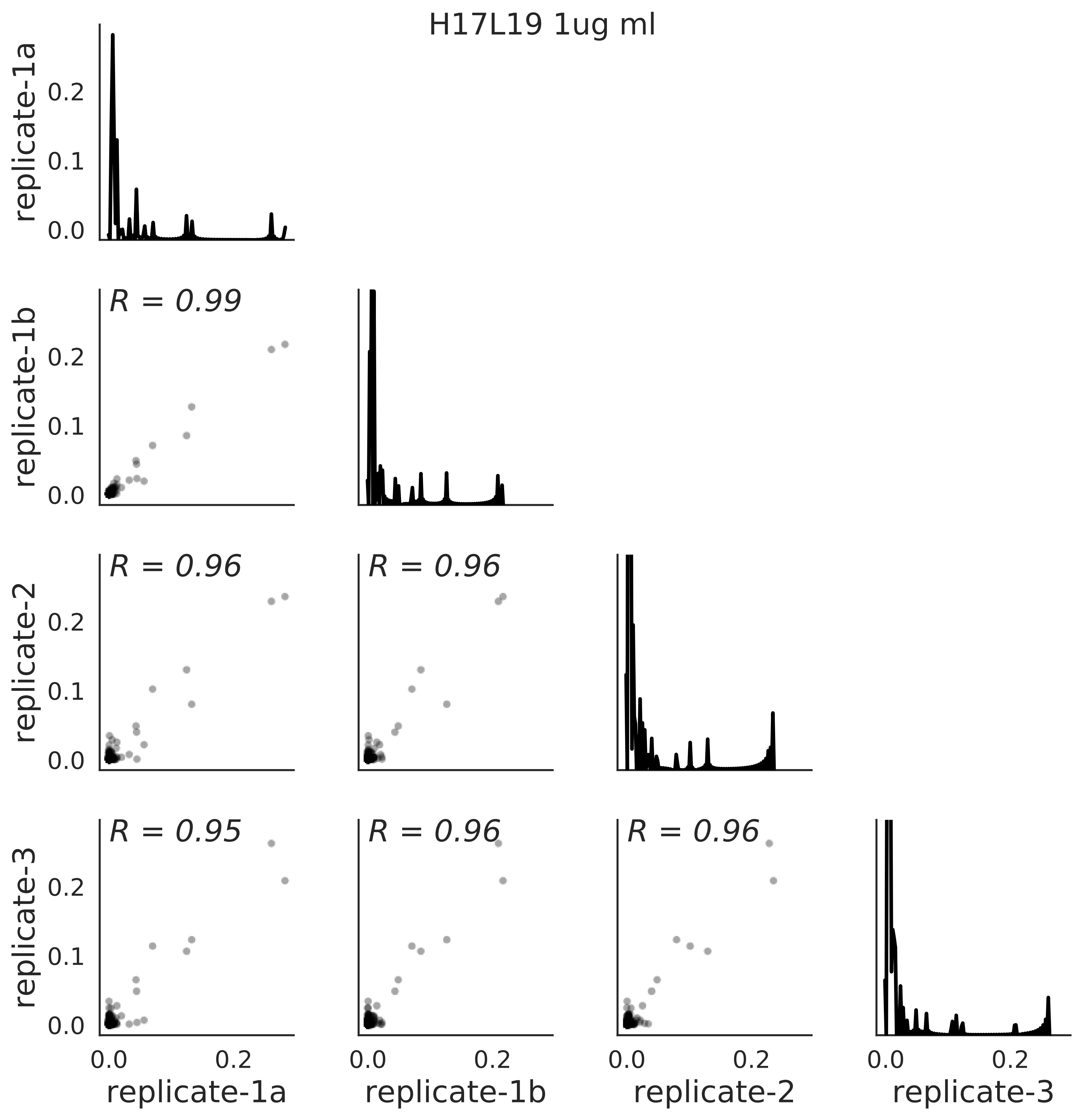

H17L19 1ug ml
replicate-1a
replicate-1b
replicate-2
replicate-3
R = 0.99
R = 0.96
R = 0.96
R = 0.95
R = 0.96
R = 0.96
0.0
0.1
0.2
replicate-1a
replicate-1b
replicate-2
replicate-3
