# Supplementary material for: How single mutations affect viral escape from broad and narrow antibodies to H1 influenza hemagglutinin
Source: Nat Commun. 2018 Apr 11;9:1386. doi: 10.1038/s41467-018-03665-3 (PMC5895760; doi:10.1038/s41467-018-03665-3)
Supplement: Supplementary file 5 — Supplementary Data 3(ZIP 19513 kb) [file 41467_2018_3665_MOESM5_ESM.zip › analysis_code/results/fracsurviveaboveavg/summary_medianavgfracsurvive.pdf]

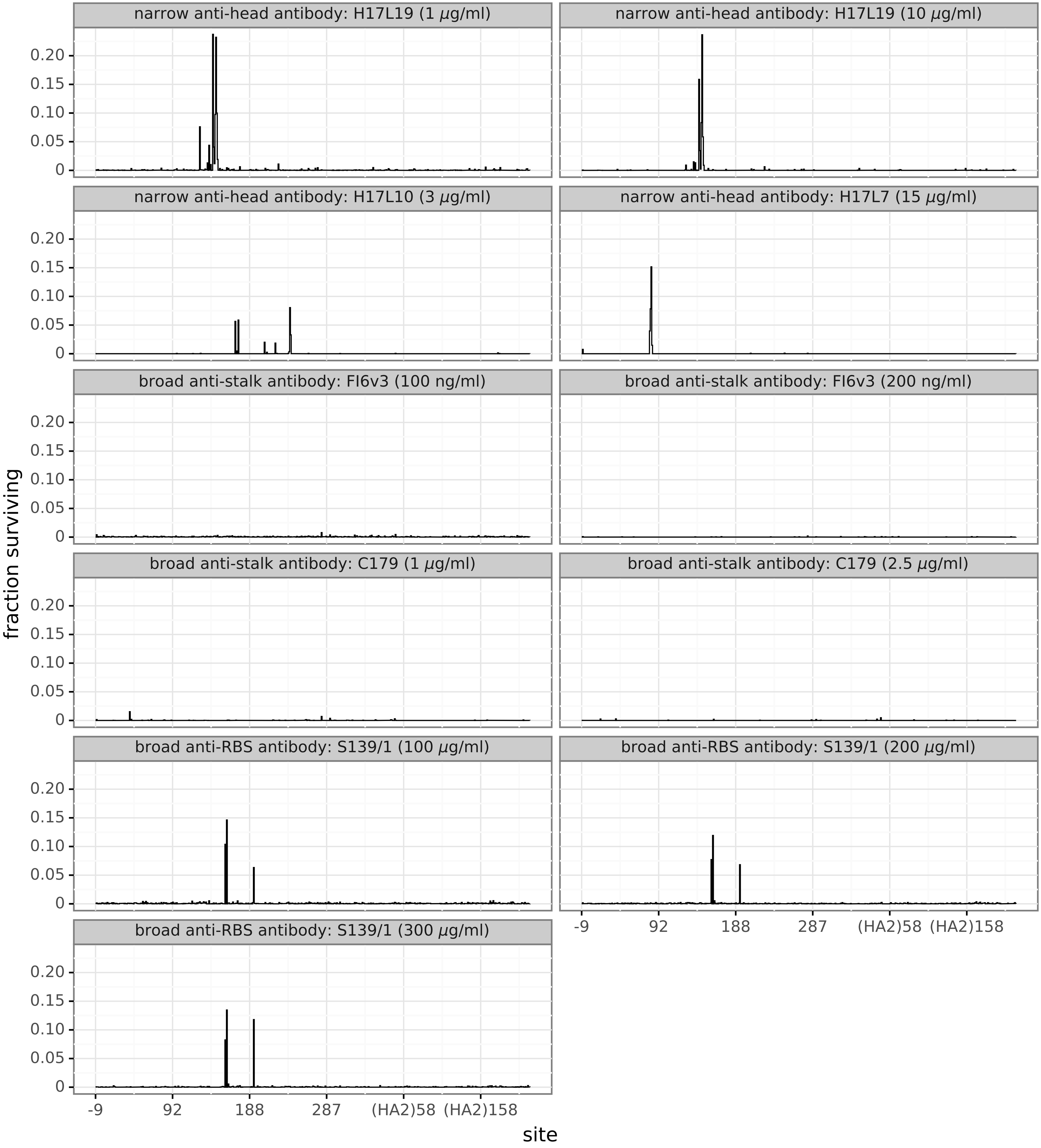
narrow anti-head antibody: H17L19 (1 μg/ml)
narrow anti-head antibody: H17L19 (10 μg/ml)
narrow anti-head antibody: H17L10 (3 μg/ml)
narrow anti-head antibody: H17L7 (15 μg/ml)
broad anti-stalk antibody: FI6v3 (100 ng/ml)
broad anti-stalk antibody: FI6v3 (200 ng/ml)
broad anti-stalk antibody: C179 (1 μg/ml)
broad anti-stalk antibody: C179 (2.5 μg/ml)
broad anti-RBS antibody: S139/1 (100 μg/ml)
broad anti-RBS antibody: S139/1 (200 μg/ml)
broad anti-RBS antibody: S139/1 (300 μg/ml)
0.20
0.15
0.10
0.05
0
-9
92
188
287
(HA2)58
(HA2)158
fraction surviving
site
