# Supplementary material for: How single mutations affect viral escape from broad and narrow antibodies to H1 influenza hemagglutinin
Source: Nat Commun. 2018 Apr 11;9:1386. doi: 10.1038/s41467-018-03665-3 (PMC5895760; doi:10.1038/s41467-018-03665-3)
Supplement: Supplementary file 5 — Supplementary Data 3(ZIP 19513 kb) [file 41467_2018_3665_MOESM5_ESM.zip › analysis_code/results/prefs/WSNprefs-H3numbering_prefs.pdf]

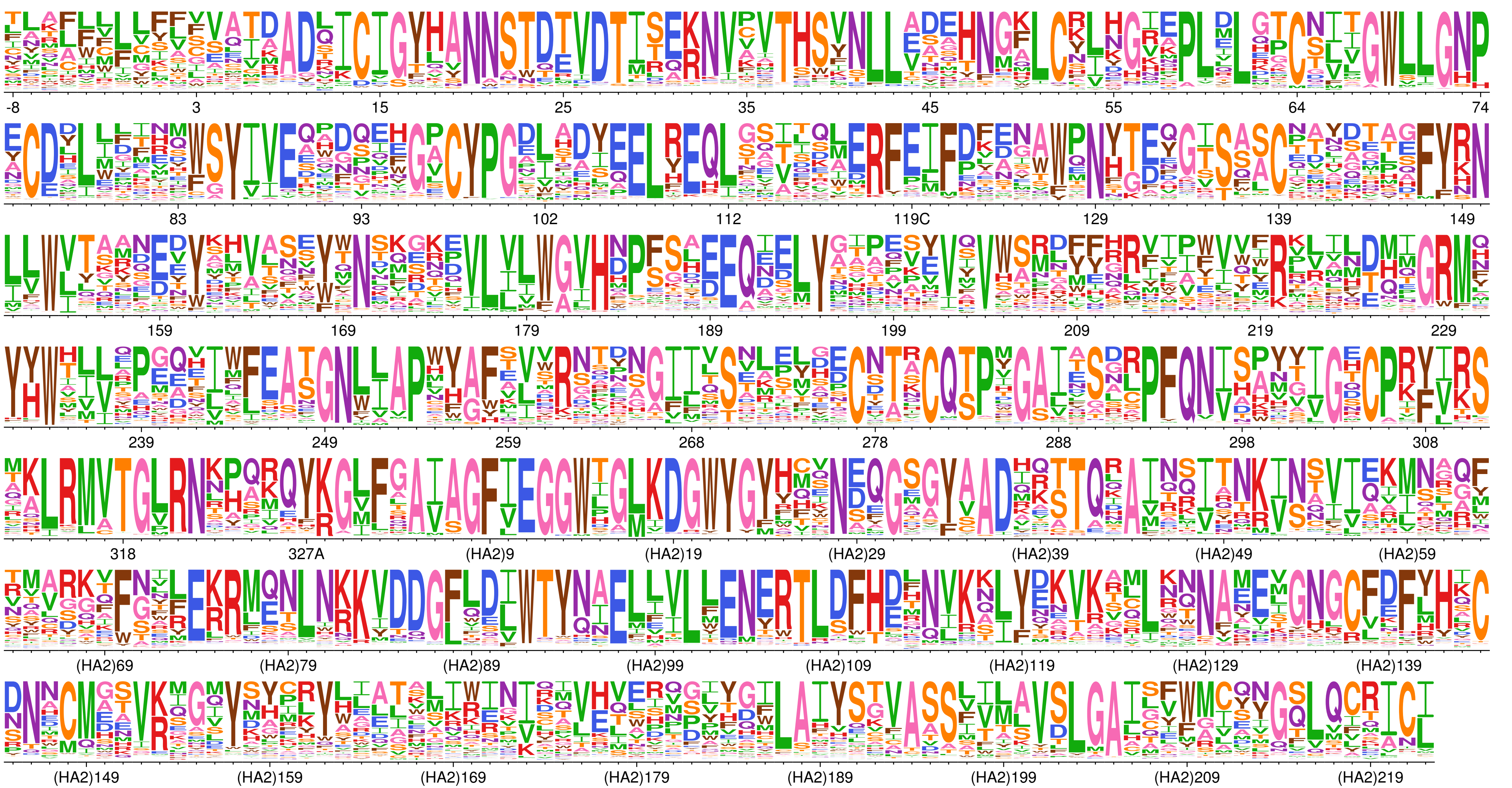
-8 3 15 25 35 45 55 64 74
83 93 102 112 119C 129 139 149
159 169 179 189 199 209 219 229
239 249 259 268 278 288 298 308
318 327A (HA2)9 (HA2)19 (HA2)29 (HA2)39 (HA2)49 (HA2)59
(HA2)69 (HA2)79 (HA2)89 (HA2)99 (HA2)109 (HA2)119 (HA2)129 (HA2)139
(HA2)149 (HA2)159 (HA2)169 (HA2)179 (HA2)189 (HA2)199 (HA2)209 (HA2)219
